# 1-(2,5-Dimethoxy-4-iodophenyl)-2-aminopropane (DOI): From an Obscure to Pivotal Member of the DOX Family of Serotonergic Psychedelic Agents – A Review

**DOI:** 10.1021/acsptsci.4c00157

**Published:** 2024-05-08

**Authors:** Richard A. Glennon, Małgorzata Dukat

**Affiliations:** Department of Medicinal Chemistry School of Pharmacy, Virginia Commonwealth University, Richmond, Virginia 23298, United States

**Keywords:** classical hallucinogens, serotonin receptors, 5-HT_2_ receptor agonists, radioligand binding, depression

## Abstract

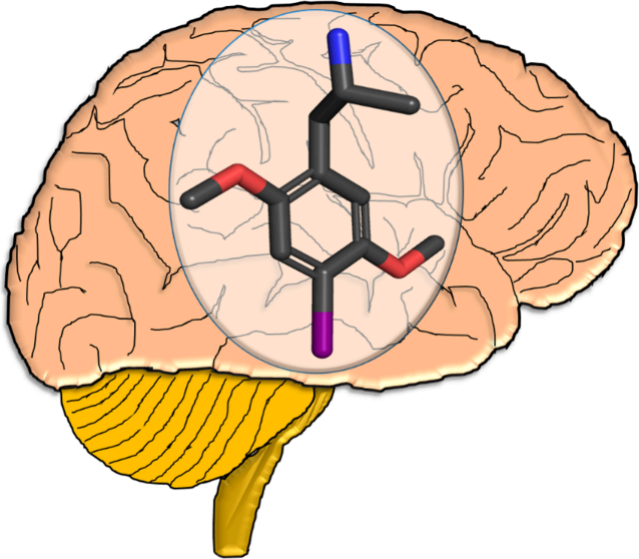

1-(2,5-Dimethoxy-4-iodophenyl)-2-aminopropane (DOI, or DOX where X = −I) was first synthesized in 1973 in a structure–activity study to explore the effect of various aryl substituents on the then newly identified, and subsequently controlled, hallucinogenic agent 1-(2,5-dimethoxy-4-methylphenyl)-2-aminopropane (DOM, or DOX where X = −CH_3_). Over time, DOI was found to be a serotonin (5-HT) receptor agonist using various peripheral 5-HT receptor tissue assays and later, following the identification of multiple families of central 5-HT receptors, an agonist at 5-HT_2_ serotonin receptors in rat and, then, human brain. Today, *classical hallucinogens*, currently referred to as *serotonergic psychedelic agents*, are receiving considerable attention for their potential therapeutic application in various neuropsychiatric disorders including treatment-resistant depression. Here, we review, for the first time, the historical and current developments that led to DOI becoming a unique, perhaps a landmark, agent in 5-HT_2_ receptor research.

There is currently a renewed and growing interest in *serotonergic psychedelic agents* or *classical hallucinogens* for use in various neuropsychiatric disorders including treatment-resistant depression (TRD).^e.g.^^1−10^ A broad structural and mechanistic variety of antidepressants are currently being explored but, as a group, serotonergic psychedelic agents represent a large class of agents with psychotherapeutic potential.^11^ In fact, recognizing their potential therapeutic utility, the U.S. Food and Drug Administration has recently published recommended guidelines for the investigation of such agents.^12^ These agents can be broadly divided into two large structural classes: (i) indolealkylamines, exemplified by *N*,*N*-dimethyltryptamine-related (or DMT-related) analogues and lysergic acid diethylamide (LSD) that possess a tryptaminergic backbone, and (ii) phenylalkylamines (see below for examples); together, these agents can be collectively referred to as arylalkylamines.^13^ Nearly all of the recent literature on the psychotherapeutic potential of arylalkylamines has focused on indolealkylamines. Yet, phenylalkylamines, agents that include phenylethylamines, phenylisopropylamines, and other structurally related agents, represent an even larger class of compounds.

An interesting member of the psychoactive phenylalkylamine category lurking in the shadows of other better-known members of this class is the phenylisopropylamine 1-(2,5-dimethoxy-4-iodophenyl)-2-aminopropane (occasionally referred to as 2,5-dimethoxy-4-iodoamphetamine) or DOI (**1**; Figure 1). Iodo-containing therapeutic agents are a rarity among clinically approved drugs.^14^ Interestingly, and rather oddly, many more psychoactive or so-called ”hallucinogenic” agents, or related psychoactive analogues investigated in animal or human structure–activity studies, bear an iodo substituent (for example, see the material below as well as the Shulgin Index^15^). DOI (**1**), although actually quite well-known nowadays from a research perspective, was once an obscure and under-appreciated member of this large family of phenylalkylamines that, over time, has had a profound impact on serotonin receptor research.

**Figure 1:**
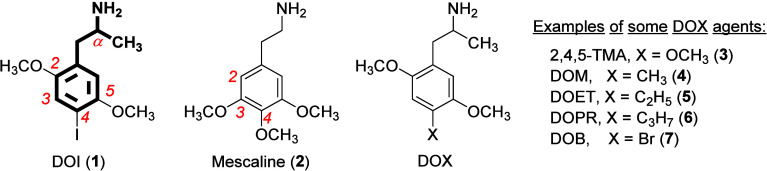
Chemical structures of several phenylalkylamines: DOI (**1**), mescaline (**2**), and some representative examples of related DOX agents: 2,4,5-TMA (**3**), DOM (**4**), DOET (**5**), DOPR (**6**), and DOB (**7**). Other DOX agents will be mentioned (*sans* structures), but they retain the 2,5-dimethoxy-4-X substitution pattern shown here. The bolded portion of DOI (**1**) represents its parent phenylisopropylamine skeleton.

This is not intended to be an exhaustive review of the DOI literature; rather, it will highlight various studies–from their earliest history to the most recent findings, including some relevant and associated salient events–that eventually made DOI a pivotal member of this family of agents. The route taken here will be in chronological order as much as possible. However, many of the types of pharmacological studies being conducted that led to what we now know about DOI and related agents have overlapping considerations. That is, there was considerable chronological overlap in the development and use of the various assay systems to investigate these agents. Initial attempts were made to develop new agents and explain the results of structure–activity studies and to relate the results of certain (presumably centrally mediated) conditioned and unconditioned behavioral assays using peripheral neurotransmitter receptor information prior to the discovery and investigation of multiple types and subtypes of brain neurotransmitter receptors using radioligand binding techniques. Studies in one discipline impacted others. For example, the results of behavioral studies and peripheral tissue assays paved the way for the later development of novel radioligands for investigating central receptors (and, here, the primary focus will be on serotonin receptors). The results of these investigations with central receptors were subsequently used to explain the findings from earlier and concurrent behavioral assays. Combined, these investigations resulted in newer agents and further investigation of DOI. Hence, the journey is somewhat circuitous and requires several digressions; however, it might provide some insight into challenges that were faced and addressed by investigators in the field over the years.

A recent PubMed search for “1-(2,5-dimethoxy-4-iodophenyl)-2-aminopropane” or “2,5-dimethoxy-4-iodoamphetamine” revealed >800 publications since 1977.^16^ Although agents belonging to what are called the *classical hallucinogens* or *serotonergic psychedelic* agents (sometimes referred to as *classical psychedelics*), we use here the terms *hallucinogen* and *serotonergic psychedelic* interchangeably to refer to these agents. The terminology associated with these agents has been discussed and distinction between these agents and *dissociative psychedelic* agents has been described. ^e.g.^(17−20) It is also quite likely that members of this large class of *serotonergic psychedelic agents* do not necessarily produce identical effects; this attribute, sometimes referred to as *polypharmacology*, implies that multiple mechanisms of action (i.e., multiple types of neurotransmitter receptors and, perhaps, other targets such as monoamine transporters and metabolic enzymes) might play a role in their actions depending upon the specific agent being considered; however, serotonergic psychedelic agents seem to share certain “*common actions*” that have allowed for their classification.

Mescaline (**2**; Figure 1), a phenylethylamine, is the oldest studied member of the hallucinogenic phenylalkylamine family; moving the methoxy substituent from the aryl 3-position to the 2-position and introduction of an α-methyl group results in a more potent hallucinogenic agent, the 2,4,5-trimethoxy analog 2,4,5-TMA (**3**; Figure 1), also referred to sometimes as TMA-2. Shulgin (for example, see page 633 in Shulgin and Shulgin)^21^ is credited with the acronymic nomenclature associated with many of these agents based, for the most part, on the structure of 2,4,5-TMA (**3**).

Structurally, DOM (**4**) can be envisioned as having arisen from 2,4,5-TMA (**3**) by removal of the 4-position methoxy group (hence, the *des*oxy or “DO” designation) and introduction of a methyl group (hence, the “M” in DOM). Accordingly, the ethyl analogue has been termed DOET (**5**), and its homologues would be named likewise, e.g. DOPR (**6**) and DOBU for the corresponding *n*-propyl and *n*-butyl compounds, respectively. Due to the incredibly large number of possible analogues, it is common to simply refer to this as the DOX series wherein the aryl 2,5-dimethoxy groups have been retained and the 4-position substituent has been varied. For example, DOHX is DOX where the 4-position substituent is an *n*-hexyl group. Obviously, DOI (**1**) is DOX where X = I; the 4-bromo counterpart of DOI is DOB (**7**). But, DOM (**4**) and LSD (**8**; Figure 2), perhaps due to their earlier appearance or enhanced potency relative to, for example, mescaline (**2**), have dominated this arylalkylamine class of agents for many years.

**Figure 2:**
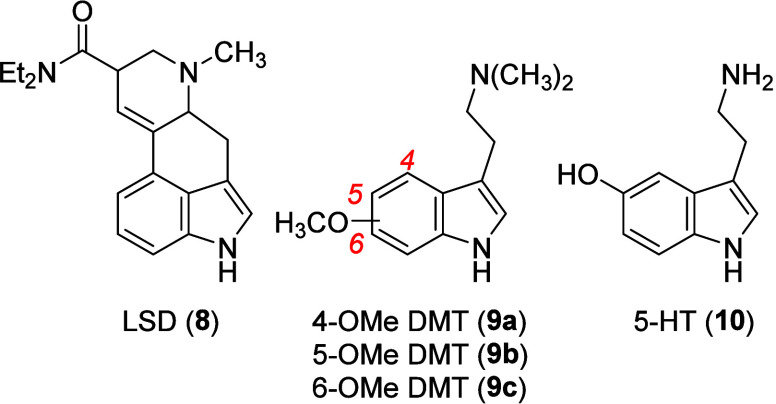
Structures of selected indolealkylamines: lysergic acid diethylamide (LSD; **8**), 4-, 5-, and 6-methoxy-*N*,*N*-dimethyltryptamine (4-, 5-, and 6-OMe DMT, **9a**-**9c**, respectively), and serotonin (5-hydroxytryptamine, 5-HT, **10**).

Both DOM (**4**) (initially referred to as “STP” – an acronym for “serenity, tranquility and peace”) and DOET (**5**), appeared on the clandestine market in the late 1960s.^22^ The earliest clinical studies were reported shortly thereafter by Snyder and colleagues^23−26^ and by Hollister et al.^27^ DOM was also included in an early human structure–activity investigation.^28^ Interestingly, Snyder and colleagues speculated that these agents might represent a potential treatment for mood disorders.^29^

The first detailed synthesis of DOM (**4**) and DOET (**5**) was described in a patent by Shulgin^30^ in 1970, and the optical isomers of 2,4,5-TMA (**3**), DOM (**4**), DOET (**5**), and DOB (**7**) were prepared soon thereafter by Nichols et al.^31^ As medicinal chemists are wont to do, the structure of DOM was manipulated to investigate its structure–activity relationships and DOI (**1**) was first synthesized in 1973 by Coutts and Malicky.^32^ Although most DOX analogs possess a chiral center at the α carbon atom resulting in a pair of optical isomers, the convention used here is that isomers will be labeled as *R*- and *S*-, but racemic mixtures will not use a prefix. For example, (±)DOI or racemic DOI will be referred to as DOI whereas its optical isomers will be identified as *R*(−)DOI and *S*(+)DOI.

Before continuing, it should be mentioned *en passant* that DOX agents might (and should) be viewed as aryl-substituted analogs of the phenylisopropylamine central stimulant 1-phenyl-2-aminopropane (i.e., amphetamine; see the bolded portion of DOI in Figure 1) or its N-monomethyl analogue (i.e., methamphetamine). As already noted above, DOI is sometimes referred to as 2,5-dimethoxy-4-iodoamphetamine; although not chemically incorrect from a trivial nomenclature perspective, the latter term is pharmacologically misleading. That is, although DOX and amphetamine/methamphetamine bear a common phenylalkylamine structural skeleton, very simple structural changes to this skeleton can dramatically alter pharmacology and attendant mechanism(s) of action.^13^ Supportive of this concept is that DOX-related agents produce behavioral effects, as will be further described below, that are distinct from those of amphetamine/methamphetamine;^33^ for example, Quinteros-Muñoz et al.^34^ showed distinct differences in the pharmacological actions of methamphetamine and DOI in rat behavioral assays. This is not to say that amphetamine and methamphetamine cannot produce a *psychotogenic* effect in humans (i.e., *amphetamine psychosis* or *paranoid-hallucinatory psychosis*) that might involve hallucinogenic episodes.^35,36^ However, amphetamine psychosis is typically associated with chronic administration of high doses of these agents and involves (primarily catecholaminergic^35−37^) mechanisms that are distinguishable from that of DOX agents described here. In addition, whereas amphetamine-like stimulants produce an increase in rodent locomotor activity, agents such as DOI and LSD typically produce a decrease.^38^ Moreover, it is the *S*-isomers of amphetamine-like stimulants (e.g., dextroamphetamine) that are more potent in animal and human studies than their *R*-enantiomers, whereas the reverse is true of phenylalkylamine hallucinogens as will be described below.

## DOX Analogs and Peripheral Serotonin Receptors

Early on, there was speculation that hallucinogenic indolealkylamines such as LSD (**8**), 4-methoxy-*N,N*-dimethyltryptamine (4-OMe DMT, **9a**), and 5-methoxy-*N*,*N*-dimethyltryptamine (5-OMe DMT, **9b**; Figure 2) might produce their effects via a serotonergic mechanism; after all, such agents bear a structural similarity to the neurotransmitter serotonin (5-HT; **10**).^e.g.^^39^ But, central 5-HT receptors had yet to be understood or even identified. From the late 1950s through the early 1980s, one of the most sensitive and commonly employed *in vitro* assays for examining the actions of 5-HT was the peripheral rat fundus preparation; various indolealkylamines were found to bind and/or act as agonists.^e.g.^^40−44^ For example, the affinities for a series of DMT analogues was 5-OMe DMT (**9b**) > 4-OMe DMT (**9a**) > 6-OMe DMT (**9c**).^45^

Such studies were latter extended to phenylalkylamines including DOX-related agents, and a variety of isolated tissue preparations were explored. For example, Cheng et al.^46^ found that DOM was active on vascular strips of dog dorsal metatarsal vein in activating 5-HT receptors in this preparation. Dyer et al.^47^ found potencies of DOB > DOET > DOM using sheep umbilical artery preparations, with *R*(−)DOM being more potent than *S*(+)DOM. The QSAR of several DOX-related analogues as agonists in the sheep umbilical artery preparation was investigated;^48−50^ although DOI was not examined, Nichols at al.^48^ introduced the idea of an optimal directional lipophilicity for the 4-position (i.e., the X of DOX) substituent as an important structural feature. In rat aortic strips, *R*(−)DOB was found more potent than racemic DOB.^51^ Contraction of guinea pig trachea by DOX agents via activation of 5-HT receptors has also been examined.^52^ The peripheral (i.e., non-CNS) actions and assays used to investigate 5-HT and various serotonergic agonists and antagonists has been reviewed.^53^

Perhaps the most extensive amount of comparative data for phenylalkylamines at peripheral 5-HT receptors is derived from isolated rat fundus strips. Huang and Ho^54^ were the first to demonstrate that DOM acts on receptors of the peripheral rat fundus preparation, and Standridge et al.^55^ found *R*(−)DOM more potent than *S*(+)DOM. DOM, DOET, and DOB were among the higher-affinity agents at rat fundus 5-HT receptors with affinities comparable to that of 5-HT itself.^54−60^

None of these studies included DOI (**1**). Subsequently, DOI was shown to bind at fundus receptors with high affinity, and *R*(−)DOI displayed somewhat higher affinity than its racemate.^61^ When a large series of DOX-related phenylalkylamines was examined at fundus 5-HT receptors, the order of affinity was DOI (**1**) > DOB (**7**) > DOET (**5**) > DOM (**4**) > 2,4,5-TMA (**3**), and for the entire series, *R*(−)DOI was the highest-affinity member (reviewed^45^). Also found was a significant correlation between rat fundus receptor affinity and hallucinogenic potency for 14 DOX-related compounds for which human data were then available.,^45,61^

## Drug Discrimination Studies

The relationship between animal drug discrimination studies and serotonin receptor action is tightly intertwined. Hence, the former will be discussed prior to turning attention to brain 5-HT receptors.

In drug discrimination studies, animals (most commonly, but not exclusively, rats) are trained to respond in one manner when administered a given dose of a *training drug* and to respond differently when administered a different drug, drug dose, or saline (i.e., *nontraining drug conditions*).^62^ For example, using a standard two-lever operant procedure, rats can be trained to reliably respond on one lever following administration of the *training dose* of a training drug, and to respond on the opposite lever when administered vehicle (e.g., saline), or even a lower dose of the training drug in a graded manner. Because the results are dose-responsive, a dose–response curve can be constructed, and an ED_50_ value can be calculated for the training drug under extinction (i.e., nonreinforcement) conditions. Adding to the usefulness of this procedure, the trained animals can be administered doses of a *test drug* (i.e., a *challenge drug)* in tests of *stimulus generalization* or *substitution* (again, under extinction conditions). If a test drug substitutes for the training drug (i.e., if some dose of the test drug results in the animals making >80% of their responses on the training drug-appropriate lever), this is taken as evidence that the test drug can produce stimulus effects in common with (but not necessarily identical to) that of the training drug. Here too, an ED_50_ dose can be calculated. In effect, the procedure can identify (i) whether or not the test drug produces stimulus effects common to the training drug–a qualitative comparison–and, if so, (ii) how potent the test drug is compared to the training drug–a quantitative comparison. The specific methodology, application, and limitations of the procedure have been reviewed.^13,62,63^

As mentioned above, certain hallucinogenic indolealkylamines such as LSD (**8**) are structurally similar to the neurotransmitter serotonin (5-HT, **10**). Hence, it was initially thought that these agents might act via a serotonergic mechanism.^39^ Because of possible serotonergic involvement in their actions, mescaline (**2**),^e.g.^^64,65^ LSD (**8**)^e.g.^^66,67^ and 5-OMe DMT (**9b**),^e.g.^^68^ as well as other hallucinogenic agents were examined as training drugs in rat drug discrimination studies. The literature on the use of hallucinogenic agents as training drugs has been reviewed.^e.g.^^13,62,66,69,70^

**Figure 3:**
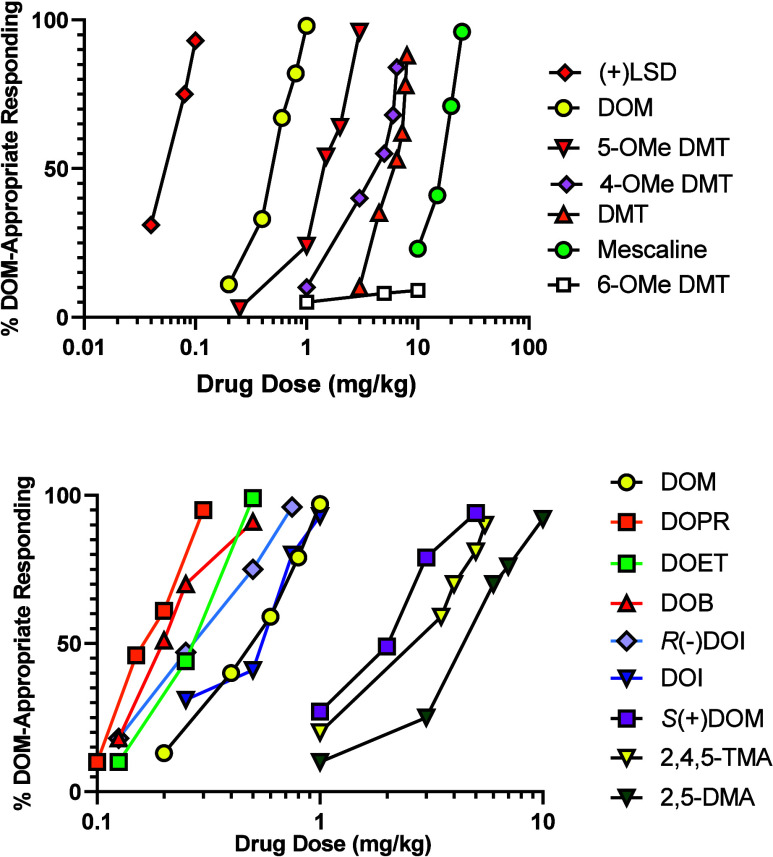
Results of stimulus generalization studies with LSD (**8**), 4-methoxy-*N,N*-dimethyltryptamine (4-OMe DMT; **9a**), 5-OMe DMT (**9b**), 6-methoxy-*N,N*-dimethyltryptamine (6-OMe DMT; **9c**), DMT, and mescaline (**2**) relative to DOM (**4**) (upper panel) and for several DOX agents (lower panel) in rats trained to discriminate DOM (1.0 mg/kg, i.p.) from vehicle. The plots were generated from tabular data provided from the same laboratory but published separately;^61,82−84^ standard errors, although reported in the cited literature, were not included here for purpose of clarity.

Winter,^64^ using the phenylethylamine mescaline (**2**) as training drug in rats, found that administration of the phenylisopropylamines DOM (**4**) and DOET (**5**) resulted in stimulus generalization. In other studies using rats trained to discriminate 5-OMe DMT (**9b**) from vehicle, the stimulus was attenuated by 5-HT antagonists, and stimulus generalization (substitution) was observed with various other indolealkylamine hallucinogens.^63,71^ In fact, there was a positive relationship between their above-mentioned rat fundus-derived 5-HT receptor affinity and stimulus generalization potency.^71^ However, although certain other nonindolealkylamine hallucinogenic agents, such as DOM (**4**) substituted in the 5-OMe DMT-trained animals, it was subsequently demonstrated that (i) DOET (**5**) and DOB (**7**) failed to substitute, and (ii) the stimulus effects of 5-OMe DMT were training-dose dependent.^72−74^ Indeed, training dose sometimes is a confounding factor in the interpretation of drug discrimination findings.^75^ It might be noted that the stimulus effects of LSD were also shown to be training-dose dependent (see White and Appel^76^ and discussion therein). Other hallucinogenic (and possible hallucinogenic) agents were investigated as training drugs. Silverman and Ho^77^ established DOM (**4**) as an effective training drug in rats, showed that its effects could be antagonized by the 5-HT receptor antagonists methysergide and cinanserin, and that stimulus generalization occurred upon administration of DOET (**5**). Later that same year, we followed this lead and trained rats to discriminate DOM from vehicle;^78^ over the course of many years, we ultimately examined (i) the structure–activity relationships of a large variety of agents, including their optical isomers (where applicable), (ii) the relationship between stimulus generalization potency and human hallucinogenic potency, and (iii) the mechanistic underpinnings of these actions. Various indolealkylamine hallucinogenic agents [e.g., 4-OMe DMT (**9a**), 5-OMe DMT (**9b**), DMT, LSD (**8**)],^79^ nonhallucinogenic agents, and “presumably” nonhallucinogenic agents were examined [e.g., there are no human data reported for 6-OMe DMT (**9c**), although even since its first synthesis, 6-OMe DMT has been found to be significantly less behaviorally active in rats than 5-OMe DMT,^80^ but human data are still unavailable]. Figure 3 shows dose–response curves for several of these indolealkylamines, and the phenylethylamine mescaline and the phenylisopropylamine DOM for comparison (upper panel), and the results with some representative DOX agents (lower panel) in DOM-trained rats. Table 1 (see later) lists ED_50_ doses for the DOX agents from Figure 3 and a few additional compounds. It might be noted that it was once thought, that because DOI substituted in DOM-trained animals, that DOI was being rapidly metabolized to 1-(2,5-dimethoxyphenyl)-2-aminopropane (2,5-DMA or DOX where X = H); this notion was dispelled when DOI was found to be >10-fold more potent than 2,5-DMA.^69^

**Table 1:** 5-HT_2_ Receptor Affinities (*K*_i_) of Some Representative DOX Agents at [^3^H]Ketanserin-Labeled Sites, Their Stimulus Generalization Potencies (ED_50_) in DOM-Trained Rats, and Human Hallucinogenic Doses for Comparison

Agent	*K*_i_ (nM)^a^	ED_50_ (mg/kg, i.p.)^b^	Human Dose (mg, p.o.)^c^
2,5-DMA	5,200	5.51	80–160
2,4,5-TMA (**3**)	1,650	3.59	20–40
DOM (**4**)	100	0.44	3–10
R(−)DOM	60	0.21	<DOM^d^
DOET (**5**)	100	0.23	2–6
DOPR (**6**)	69	0.17	2.5–5
DOB (**7**)	63	0.20	1–3
R(−)DOB	60	0.10	<DOB^d^
S(+)DOB	120	0.81	>DOB^d^
DOI (**1**)	19	0.42	1.5–3.0
R(−)DOI	10	0.26	<DOI^d^
S(+)DOI	35	1.70	>DOI^d^
DOTB^e^	19	NSG	-f
DOAM^e^	7	NSG	>10^g^
DOBZ^e^	7	NSG	-f

a Binding data from rat brain cortical homogenates.^141−143^

b Data using rats trained to discriminate DOM (1.0 mg/kg, i.p.) from vehicle.^69,83,84^ For purpose of comparison, ED_50_ values (mg/kg) for several related agents include *S*(+) DOM = 1.7, *R*(−)DOET = 0.09, *S*(+)DOET = 0.85, N-monomethyl DOM = 3.99, and LSD (**8**) = 0.05.

c Human psychoactive potency range from Shulgin and Shulgin.^21^

d Isomers were either more potent (<) or less potent (>) than their racemates, but specific dose ranges were not provided.

e DOX analogs where X = *tert*-butyl (DOTB), amyl (i.e., *n*-pentyl) (DOAM), and benzyl (DOBZ).

f Data not provided; agents not examined in detail.

g The >10 mg dose indicates that, should DOAM be active, higher doses might be required.

Almost immediately, some rank-order potency differences became obvious when comparing stimulus generalization data from 5-OMe DMT (1.5 mg/kg)-trained and DOM (1.0 mg/kg)-trained rats. For example, although LSD was the most potent agent in both group of animals, the order of potency in the 5-OMe DMT-trained animals was LSD > 5-OMe DMT > DOM > 4-OMe DMT > DMT > 6-OMe DMT > mescaline;^81^ this can be compared with the results from DOM-trained animals (Figure 3, upper panel) with the order of potency being LSD > DOM > 5-OMe DMT > 4-OMe DMT > DMT > mescaline, and where 6-OMe DMT produced saline-appropriate responding at the highest doses examined. A number of other agents were compared in both groups of animals, but only a few representative examples are provided here; for example 7-methoxy-*N,N*-dimethyltryptamine substituted in 5-OMe DMT-trained animals (with lower potency than 6-OMe DMT) but did not substitute in DOM-trained animals.^81^ Evidently, the 5-OMe DMT stimulus, although perhaps somewhat similar to the DOM stimulus, was most likely acting via a somewhat different or more complex receptor-mediated mechanism.

Of note is that there are six possible trimethoxy positional isomers of phenylisopropylamine, with one of them being 2,4,5-TMA (**3**); of these, five have been examined and all substituted in DOM-trained animals with 2,4,5-TMA (ED_50_ = 3.59 mg/kg) being among the most potent. The other analogues (followed by ED_50_ value) include 2,4,6- (3.69 mg/kg), 3,4,5- (or α-methyl mescaline; 6.34 mg/kg), 2,3,4- (7.8 mg/kg) and 2,3,5-trimethoxyphenylisopropylamine (16.5 mg/kg).^33^ As mentioned in the Introduction, DOX agents are sometimes referred to as substituted amphetamines, but produce behavioral effects distinct from the latter. Neither DOM, *R*(−)DOM, *S*(+)DOM, DOET, nor any of the trimethoxy substituted phenylisopropylamines (e.g., 2,4,5-TMA) substituted in rats trained to discriminate (+)amphetamine from vehicle,^33,77,85,86^ and neither amphetamine nor methamphetamine substituted in DOM-trained rats.^86^ Also, there are four DOX analogues that bear a halogen substituent at the 4-position (i.e., DOX where X = -I, -Br, -Cl, -F); that is, in addition to DOI (**1**) and DOB (**7**), there are the 4-chloro (i.e., DOC) and 4-fluoro (i.e., DOF) analogues. All four halogenated DOX analogues substituted in DOM-trained rats with an order of potency, on a μMole/kg basis, of DOB > DOI ≅ DOC > DOF.^85^ Rather than providing an extensive compilation of the large number of agents examined and a detailed structure–activity account here, such information has been summarized elsewhere.^e.g.^^69,70,87−89^ The discrimination-derived structure–activity relationships formulated for psychoactive arylalkylamines in DOM-trained rats can be compared with the structure–activity relationships derived/formulated from various other animal assays and human studies,^28,90−92^ and are usually quite consistent depending upon the particular assay system (but, see later). QSAR or quantitative structure–activity relationship studies, to shed light on how physicochemical properties of various substituents might contribute to a given action, are rarely (almost never) conducted using *in vivo* data due to potential problems associated with absorption, distribution, and/or metabolism when varying structure types are compared. However, for a series of DOX agents varying in structure only by alteration of their X substituent, which might be viewed as a “*matched set*”, a relating equation was obtained (i.e., Log 1/ED_50_ = 2.28π – 0.94π^2^ + 4.81; n = 11, r = 0.977) in which substitution potency (ED_50_) in DOM-trained animals was shown to be related to lipophilicity: π and π^2^ of the 4-position substituent.^89^ Occurrence of both π and π^2^ terms in the equation reflects a parabolic relationship, with a maximum value related to the negative contribution of π. That is, potency increased as the lipophilicity (π value) of the X substituent increased, but decreased once an optimal lipophilicity was achieved (as determined from the negative contribution of the π^2^ term to the relating equation). DOI (**1**), 2,4,5-TMA (**3**), DOM (**4**), DOET (**5**), DOPR (**6**), DOB (**7**), DOC and DOF were included in the study.^89^ Involvement of central 5-HT_2_ receptors in the actions of DOX-type agents, and the relationship between drug discrimination findings and human data will be discussed below.

## Unconditioned Behavior

Hallucinogenic agents can elicit several conditioned and unconditioned behaviors in animals; one of the most popular unconditioned behaviors for investigation of these agents is the rodent head-twitch response (HTR),^93^ sometimes referred to as head-shake behavior, that consists of rapid side-to-side rotational head movements (reviewed^94−97^). This behavior is exhibited by rats and, more commonly, mice after administration of serotonergic hallucinogens and certain other 5-HT receptor agonists. The HTR is not limited to hallucinogenic agents; but, where serotonergic agents have been examined, this effect has been blocked by a variety of 5-HT antagonists including ketanserin and pirenperone^94^ (see below for further discussion on these two antagonists). DOI produced the mouse HTR^e.g.^^98−101^ with *R*(−)DOI being twice as potent as *S*(+)DOI, and the response was antagonized by ketanserin.^98,99^ A single dose of DOI resulted in tolerance to the DOI-induced HTR at 24 h postinjection but in supersensitivity at 48 h suggesting that the serotonergic system adapts to chronic exposure of agonists (and antagonists).^102,103^ DOI also produced the HTR in rats, and the effect, although 5-HT_2_-mediated, was found to be modulated by 5-HT_1A_ as well as D_1_ and D_2_ dopamine receptor involvement.^104^ The HTR in mice and rats using DOI and other agents has been compared.^105^ The shrew (*Cryptotis parva*) seems more sensitive than mice to agents producing the HTR, and DOI was found effective in this species.^106^

Another assay used to examine DOX-related agents, although considerably less data are available than from the HTR assay, is the mouse ear-scratch response (ESR),^107^ and the effect can be blocked with 5-HT antagonists.^95^ The ESR is produced by DOM and DOET, but not by nonhallucinogenic phenylalkylamines, and appears to be stereoselective with the *R*(−)-enantiomers of active agents being the more potent.^107,108^ DOI produced the ESR and *R*(−)DOI was about six times more potent that its *S*(+)-enantiomer; the DOI effect was blocked by very low doses of ketanserin.^109^

A caveat is that the DOI-elicited HTR in mice is seemingly a 5-HT_2A_ agonist-mediated effect, but with possible modulation of the behavior that might involve a competing 5-HT_2C_ agonist component;^110−112^ more will be discussed about these 5-HT receptors in the next section. Some have recently argued that activation of 5-HT_2C_ receptors, alone or in concert with 5-HT_2A_ receptor activation, yields comparable HTR behavior involving divergent receptor-mediated downstream signaling.^113^ Although the specific signaling cascades mediating the HTR have not been conclusively identified, Gq and β-arrestin2 have been implicated. Recent studies with different existing and novel agents, including DOI, found that the HTR was correlated with Gq efficacy but not with β-arrestin2 recruitment.^114^

In any event, the HTR behavior in mice has now become a rather routine application. Many have since examined the action of DOI on this behavior and have provided additional information and insight involving drug interactions, testing parameters and conditions, and novel methodologies. Although not a comprehensive listing, HTR studies involving DOI are becoming ever-increasingly used.^e.g.^^115−125^ Notably, Halberstadt et al.^126^ have demonstrated that a significant correlation exists between the mouse HTR and the results of published drug discrimination studies with serotonergic hallucinogens using rats trained to discriminate either DOM or LSD from vehicle; a large number of agents was examined including DOM, DOET, DOB, and *R*(−)DOI. Given the reduced cost and labor associated with the HTR using mice relative to drug discrimination studies with rats, the former will probably see increased application in the future.

Mechanistically, 5-HT_2_ receptor agonism is inarguably involved in the mouse HTR produced by hallucinogenic agents. This is a given, and this has been demonstrated time and time again. However, administration of the mGlu2/3 agonist LY354740 suppressed the HTR induced by DOI, whereas administration of the mGlu2/3 antagonist LY341495 enhanced the frequency of DOI-induced HTR, raising the possibility that the actions of DOI might be mediated, in part, via increased glutamate release.^125,127^ Another study also found that the selective mGlu2/3 agonists LY354740 and LY379268 inhibited DOI-induced HTR in mice.^128^ It has been suggested that Group II metabotropic glutamate receptor agonists are capable of modulating postsynaptic function preferentially in the limbic cortex under conditions of enhanced glutamate release.^129,130^

## Central Serotonin Receptors

The year 1979 represented a turning point for serotonin receptor research in that, after quite a few years of investigation by several investigators, 5-HT_1_ and 5-HT_2_ receptors were identified in mammalian brain.^131^ Multiple families or populations of central 5-HT receptors have since been identified and these are referred to as 5-HT_1_-5-HT_7_ serotonin receptors with most, but not all, being G-protein-coupled (i.e., 5-HT_3_ receptors are ligand-gated ion channel receptors) (reviewed^132^). Today, three subpopulations of G-protein coupled 5-HT_2_ receptors are recognized: 5-HT_2A_, 5-HT_2B_ (initially termed 5-HT_2F_ receptors because they were first identified in the peripheral rat fundus), and 5-HT_2C_ receptors (initially termed 5-HT_1C_) receptors. Earlier studies showing a correlation between rat fundus receptor affinity and human hallucinogenic potency was becoming clearer in that fundus serotonin receptors were now considered members of the 5-HT_2_ receptor family. An early investigation using [^3^H]ketanserin and [^3^H]mesulergine to label rat 5-HT_2A_ and 5-HT_2C_ receptors, respectively, showed that for 34 DOX-related agents (including DOI, 2,4,5-TMA, DOM, DOET, DOPR and DOB) there was a significant correlation (r > 0.9) between their *K*_i_ values.^133^ In fact, some years later, a significant correlation was demonstrated between human 5-HT_2A_ and both human 5-HT_2B_ and human 5-HT_2C_ receptor affinities.^134^ The 5-HT_2A_ and 5-HT_2B_ functional activity of a series of DOX-related agents (including DOI) has been compared.^135^ A binding profile for DOI at some common receptors has been published.^136^

Digressing here, once 5-HT_1_ and 5-HT_2_ populations of brain 5-HT receptors were identified, many investigators working with peripheral 5-HT receptor tissue preparations at the time shifted their attention to these CNS receptors using rat brain cortical homogenates and, subsequently, human brain homogenates and, even later, to cloned rat and human receptor preparations. It was generally agreed that rat cortical 5-HT_2_ receptors represented the rat version of what was later termed human 5-HT_2A_ receptors. In the mid to late 1980s, new 5-HT receptor types were still being identified (originally termed *binding sites* in the early literature but now termed *receptor populations or subpopulations*); 5-HT_1_, 5-HT_2_, and 5-HT_3_ receptors had been proposed, and subpopulations of 5-HT_1_ receptors were beginning to be reported. The selectivity of various agents for the different 5-HT receptor populations/subpopulations was a major and rather vexing problem at the time in that recognized 5-HT agonists (and antagonists), as well as newly developed serotonergic agents, required continual re-evaluation each time a new 5-HT receptor population was identified.^137^ Moreover, given that many investigators were attempting to develop or identify *in vitro* and *in vivo* functional assays for various serotonergic agents, and to relate them to activation or antagonism of the different 5-HT receptor populations, this became a nearly Sisyphean chore.^138^ In general, however, although some exceptions exist,^e.g.^^139^ the greater the structural similarity between a serotonergic agent and that of 5-HT, the lower its selectivity.^140^ For example, various indolealkylamines displayed affinity for 5-HT_1_ and 5-HT_2_ receptors; however, DOX-related and certain other phenylalkylamines displayed higher affinity for 5-HT_2_ receptors. This made DOX agents–compounds lacking an indolealkylamine moiety–attractive targets for further examination of selectivity. One of the highest-affinity members of the DOX/phenylalkylamine family at 5-HT_2_ receptors was DOB (**7**) (*K*_i_ = 63 nM);^141^ the DOB structure was “*deconstructed*” and it was found that removal of any of the aryl substituents (i.e., the bromo group, or either one or both of the methoxy groups, or a combination thereof) resulted in a substantial (i.e., 500- to >1,000-fold) decrease in affinity, and that the affinity of *R*(−)DOB was higher than that of *S*(+)DOB. Consequently, DOI (**1**) was examined in greater detail. Because *S*(+)DOI had not been previously reported, it was synthesized for comparison with *R*(−)DOI, and *S*(+)DOI (*K*_i_ = 35 nM) displayed only slightly lower affinity than did *R*(−)DOI (*K*_i_ = 10 nM).^141,142^ In other words, binding was stereoselective, not stereospecific. See Table 1 for some representative binding data.

**Figure 4:**
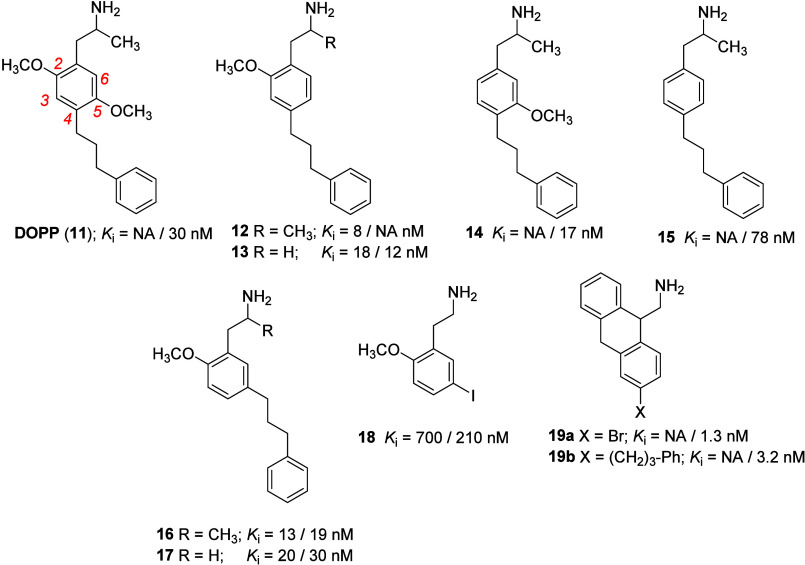
Structures and 5-HT_2A_ receptor binding data for some representative examples of DOX-related compounds with a lipophilic aryl substituent X at the 4- or 5-position, and antagonist **19** (see text). Where two *K*_i_ values are provided; the first was obtained (where data were available) using [^3^H]DOB as radioligand and the second using [^3^H]ketanserin. See text for discussion. NA = data not available.

A large number of DOX-related phenylalkylamines was examined and structure–activity and QSAR studies were conducted.^51,144^ Although 5-HT_2_ receptor affinity could be accounted for by the lipophilic and electronic character of the 4-position substituent of DOX compounds, affinity and agonist action were not synonymous. That is, some high-affinity DOX analogues with sterically large/extended 4-position substituents unexpectedly resulted in antagonist action. For example, where the X substituent was benzyl (DOBZ, *K*_i_ = 7 nM), *n*-hexyl (DOHX, *K*_i_ = 2.5 nM), or *n*-octyl (*K*_i_ = 3 nM)] the compounds acted as antagonists, whereas DOM (**4**), DOET (**5**), DOPR (**6**), DOB (**7**), and DOI (**1**) displayed agonist action in a 5-HT_2_-mediated inositol phosphate assay.^51,145^ It would appear that there is a “sweet spot” for agonist action with compounds such as DOI and DOB (as well as DOET and DOPR) bearing 4-position substituents being among the optimal. In contrast, DOPP (**11**; Figure 4) is a high-affinity 5-HT_2A_ receptor antagonist,^51^ and the 2,5-dimethoxy substitution pattern of DOX compounds was not required for high affinity.

Today, there are a variety of high-affinity nonarylalkylamine chemotypes with 5-HT_2_ antagonist action. However, the finding that certain DOX compounds acted as antagonists was unexpected. Later studies found that either one of the two methoxy groups of DOPP (**11**) could be removed without untoward effect on affinity (e.g., **12**-**14**; Figure 4);^146^ in contrast, removal of the 5-methoxy group of DOI resulted in a 10-fold decrease in affinity.^133^ Also, removal of either the 2- or 5-methoxy group in a series of DOB-related agents resulted in a substantial decrease in 5-HT_2A_ receptor affinity and in a (although lesser) decrease in potency in the mouse head-twitch assay.^147^ However, relocation of the methoxy substituents of DOPP actually enhanced affinity by as much as an order of magnitude. For example, the 2,3-, 3,5-, and 2,6-dimethoxy positional isomers of DOPP (**11**) bind with *K*_i_ values of 3–4 nM. In fact, the presence of a methoxy group is not essential for binding (i.e., **15**).^146,148^ This is in contrast to the structure–activity findings with DOB and DOI where both methoxy groups are nearly essential for binding, and that the parent structure of **15** (that is, the simplest phenylisopropylamine, amphetamine; see bolded portion of **1** in Figure 1) lacks 5-HT_2_ receptor affinity (*K*_i_ > 40,000 nM).^141^ Furthermore, transposition of the 4-[3-(phenyl)propyl] group of **12** and **13** to the 5-position (i.e., **16** and **17**) had little effect on affinity. The iodo counterpart of **17** (i.e., **18**) displayed considerably reduced affinity. Where these high-affinity compounds were evaluated, they acted as antagonists.^148^ It might be noted that removal of the two methoxy groups of DOI (**1**) results in an agent, 4-iodoamphetamine, that acts as a serotonin transporter reuptake inhibitor.^149^ These results supported the concept, as mentioned above, that *high affinity at 5-HT*_*2A*_*receptors for DOX-like compounds does not equate with agonist action*, and indicates that there appear to be differences in the structure–activity relationships of phenylalkylamine agonists and antagonists.^148^ In addition, where both [^3^H]DOB and [^3^H]ketanserin were used as radioligands in binding studies, it might be noted that these compounds showed minimal differences in affinity indicative of antagonists (see below discussion on the use of agonist radioligands such as [^3^H]DOB to label 5-HT_2_ receptors).

Structural modification of **15** led to a series of novel 5-HT_2A_ antagonists **19** (Figure 4) and when X = -Br (**19a**; *K*_i_ = 1.3 nM) or −CH_2_CH_2_CH_2_Ph (**19b**; *K*_i_ = 3.2 nM)^150^ this once again showed the independence of binding on the presence of the DOX-type methoxy-group substitution pattern. Indeed, subsequent site-directed mutagenesis studies suggested that these agents (i.e., **19**) bind differently at 5-HT_2_ receptors than do DOX agonists.^150,151^

Further suggestive of 5-HT_2_ receptor agonist affinity as underlying the behavioral effects of DOX analogs are the results of drug discrimination studies using rats trained to discriminate DOM from vehicle.^87^ Also, high-affinity agonists are among the agents found to be psychoactive in humans (see Table 1). In general, the *R*(−)-isomers typically bind at 5-HT_2_ receptors with higher affinity than their opposite enantiomers, and are the more potent in drug discrimination studies.

## DOX Compounds as Radioligands

Shortly following the discovery of brain 5-HT_1_ and 5-HT_2_ receptors, it was found that most serotonin antagonists available at the time generally bind with high(er) affinity for the latter and low(er) affinity for the former; the converse was true for serotonin agonists (e.g., for serotonin itself). For a while, it was thought that 5-HT_1_ receptors might represent the site of action of agonists and 5-HT_2_ receptors were the targets of antagonists. What was required at the time was an example(s) of a 5-HT_2_ receptor agonist. Furthermore, brain 5-HT_2_ receptors were being labeled using antagonist radioligands (e.g., radiolabeled spiperone and, later, ketanserin). To support or refute the thinking at that time, there was a pressing need to identify 5-HT_2_ receptor agonists, and 5-HT_1_ receptor antagonists. On the basis of earlier studies with peripheral 5-HT receptor preparations, it seemed possible that certain DOX-type compounds might represent the first examples of 5-HT_2_ receptor agonists. Subsequent evaluation of a series of DOX and related agents indicated that several DOX compounds displayed high affinity for rat brain 5-HT_2_ receptors and acted as agonists; ^e.g.^(141) DOI, and particularly *R*(−)DOI, were among the higher affinity compounds and some data are provided in Table 1.

Subsequently, there was a flurry of interest from several laboratories to develop an agonist radioligand for labeling 5-HT_2_ receptors, and [^3^H]DOB was developed as the first agonist radiolabel for 5-HT_2_ receptor binding studies (Table 2).^152,153^ Soon thereafter, [^77^Br]-*R*(−)DOB was examined as a radioligand for labeling human cortical 5-HT_2A_ receptors.^154^

**Table 2:** Representative 5-HT_2_ Receptor Binding Data from Rat Brain Homogenates Using a Labeled Agonist (DOB) and Antagonist (ketanserin) as Radioligand^a^

	*K*_i_ (nM)		
	[^3^H]DOB as Radioligand	[^3^H]Ketanserin as Radioligand	Fold Difference
DOB (**7**)	0.8	41	51
R(−)DOB	0.4	24	60
S(+)DOB	2.3	146	63
DOI (**1**)	0.7	19	27
2,4,5-TMA (**3**)	81	1,250	15
DOM (**4**)	8.0	100	12
R(−)DOM	1.8	60	33
DOET (**5**)	1.5	100	67
DOPR (**6**)	0.9	69	77
5-HT (**10**)	7.8	928	119
Ketanserin	1.3	1.2	1

a Binding data are from the same laboratory but were published separately.^51,152,153,155^

Agonists (e.g., 5-HT) displayed substantially higher affinity for the agonist-labeled (i.e., [^3^H]DOB-labeled) 5-HT_2_ receptors than for the antagonist-labeled receptors (Table 2). Similar results were later obtained with [^77^Br]-*R*(−)DOB-labeled receptors; for example, DOI (IC_50_ = 0.4 nM) and DOB (IC_50_ = 0.2 nM) displayed 80-fold and 200-fold higher affinity, respectively, when compared with their affinities measured using an antagonist (i.e., [^3^H]spiperone) ragioligand.^154^ These findings resulted in greater interest to develop other DOX compounds as radioligands. Several laboratories joined the fray. We^156^ prepared and evaluated [^125^I]DOI as a radioligand (Table 3) that eventually became commercially available, and Nichols and co-workers^157^ prepared both optical isomers of [^125^I]DOI. Due to the higher specific activity of ^125^I over tritium, radioiodinated compounds could be particularly useful in autoradiographic studies. McKenna and Peroutka^158^ examined the binding of a number of agents using [^125^I]-*R*(−)DOI (see Table 3). Here too, as with [^3^H]DOB as radioligand, agonists (as opposed to antagonists) tended to display enhanced affinity relative to their affinity measured using [^3^H]ketanserin as radioligand, and the *R*-isomers represented the eutomers.

**Table 3:** Affinities of Selected Agents for Rat Cortical 5-HT_2A_ Receptors Labeled with Different Radioligands

	[^125^I]DOI^a^ (*K*_i_, nM)	[^3^H]Ketanserin^a^ (*K*_i_, nM)	Fold Difference^b^	[^125^I]-*R*(−)DOI^c^*K*_i_, nM)
*R*(−)DOM	-d	-		4.8
*R*(−)DOET	-	-		0.54
*R*(−)DOB	0.4	25	62.5	0.4
*S*(+)DOB	-	-		1.0
DOI (**1**)	0.7	20	28.5	-
*R*(−)DOI	-	-		0.53
*S*(+)DOI	-	-		0.89
LSD (**8**)	-	-		3.5
5-HT (**10**)	6.1	600	98.4	3.0
Ketanserin	1.3	1.2	1.0	1.2

a Data from Glennon et al.^156^

b Agonists exhibited higher affinity for sites labeled by the agonist radioligand over the antagonist radioligand by x-fold.

c Data from McKenna et al.^158,159^

d Data not reported in the cited studies.

Using [^125^I]-*R*(−)DOI as radioligand, McKenna and Peroutka^158^ suggested that it labels a 5-HT receptor (that they tentatively designated 5HT_2A_ receptors) in rat cortex that is either absent or minimally present in bovine cortex. In contrast, [^3^H]ketanserin labeled both the putative 5-HT_2A_ site in rat cortex as well as a separate and distinct recognition site present both in rat and bovine cortex that they tentatively designated “5-HT_2B_ sites”.^158^ This led to some controversy referred to as the “*two-site versus two-state*” hypothesis. In contrast to two different receptor types, some investigators argued that 5-HT_2_ receptors exist in *high-affinity* and *low-affinity* states. Subsequent studies with cloned human 5-HT_2A_ receptors by Hartig and co-workers^160^ using [^3^H]DOB, and Titeler and co-workers^156,161,162^ using [^125^I]DOI, argued for the latter and this is now accepted.^163^ That is, radiolabeled agonists label the high-affinity state of 5-HT_2A_ receptors whereas tritiated ketanserin labels both states. Today, 5-HT_2B_ receptors, initially referred to as 5-HT_2F_ receptors, are synonymous with the 5-HT_2_ receptors found in the rat fundus preparation,^132^ and are distinct from the above-mentioned “5-HT_2B_ sites”. DOI (**1**) binds at [^125^I]DOI-labeled h5-HT_2A_ receptors (*K*_i_ = 0.7 nM) with 3-fold higher affinity than at similarly labeled 5-HT_2C_ receptors (*K*_i_ = 2.4 nM) and with 30 times higher affinity than for [^3^H]5-HT-labeled h5-HT_2B_ receptors (*K*_i_ = 20 nM).^134^

**Figure 5:**
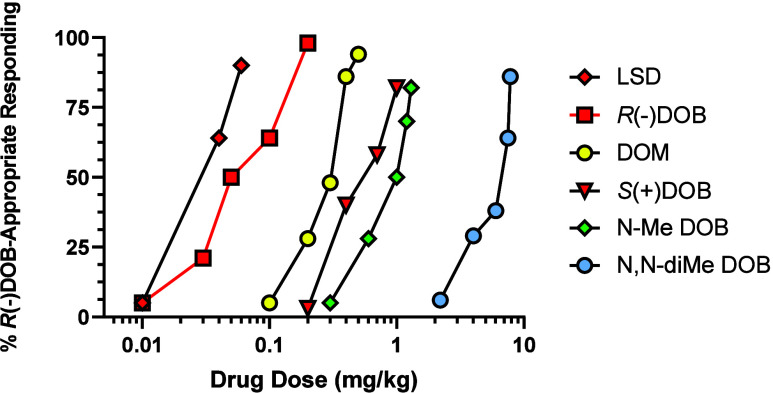
Representative results of stimulus generalization studies using rats trained to discriminate *R*(−)DOB (0.2 mg/kg, i.p.) from saline vehicle. Agents shown elicited >80% *R*(−)DOB-appropriate responding. Data are plotted primarily from tabular results that were previously reported by us.^197,198^ Although standard errors (SEM) were provided in the cited literature, they are not included here for purpose of clarity.

Autoradiographic studies have utilized [^125^I]DOI ^e.g.^(164−166) and both radiolabeled isomers.^167,168^ It should be mentioned that [^131^I]DOI and [^123^I]DOI were developed for dog and monkey imaging studies before 5-HT_2_ receptors were ever identified,^169^ and we later synthesized and examined [^123^I]DOI as a SPECT (single-photon emission computed tomography) imaging agent in Rhesus monkeys in collaboration with Dr. Kan Sam Lee (NIH)^170^ (and see Figure 7–5 in reference (62)). However, although [^123^I]-*R*(−)DOI displayed good brain uptake and localized in serotonergic areas of baboon brain, its target to nontarget ratio and its relative insensitivity to ketanserin displacement suggested high nonspecific uptake limiting its usefulness for 5-HT_2_ receptor imaging studies by SPECT.^171^

## DOX Agents, Drug Discrimination, and 5-HT_2A_ Serotonin Receptors

Prior to 1979, a variety of 5-HT receptor agonists and antagonists had been identified, mostly from earlier peripheral tissue studies, and were available for pharmacological investigation. With the subsequent identification of an ever-growing list of multiple brain binding sites as potential 5-HT receptors, it was becoming increasingly difficult to associate a particular physiological response or pharmacological action with one 5-HT receptor population over another, or the involvement of several types of 5-HT receptors, as being involved in the mechanism of action of serotonergic agents.^e.g.^^172,173^ With respect to drug discrimination studies to investigate hallucinogens, a variety of agents were examined as training drugs,^174^ and the results were, initially, quite confusing. A good example of this problem is our use of the 5-HT receptor agonist and hallucinogenic agent 5-OMe DMT (**9b**).^175^ Depending upon the training dose (using rats trained to discriminate various i.p. training doses of between 1.0 and 3.0 mg/kg), the 5-OMe DMT stimulus was blocked by certain recognized (at the time) 5-HT antagonists but not by others, and substitution occurred with certain DOX agents but not with some that had previously shown agonist action in peripheral (see above) tissue assays. It was speculated that 5-HT_1_ receptor agonism might be playing a role in the 5-OMe DMT stimulus depending upon its training dose,^175^ and Spencer et al.^176^ later demonstrated, using rats trained to a low dose of 5-OMe DMT (1.25 mg/kg) that, at this dose, its stimulus effects were primarily mediated by 5-HT_1A_ receptors but involved a 5-HT_2_ receptor component. Since that time, the 5-OMe DMT stimulus has been suggested to be *nonselective* between 5-HT_2_ and 5-HT_1A_ receptors (depending upon the training dose),^177^ and was later abandoned. Hence, a different “hallucinogen” would be required to further investigate the stimulus effects of other hallucinogenic agents.

Eventually, we found that the discriminative stimulus effect of DOM as a training drug in rats was not dose-dependent, was attenuated by pretreatment of the animals with certain 5-HT antagonists (later found to be somewhat 5-HT_2_ receptor “selective”), but not by other less-selective antagonists, and that DOM-stimulus generalization failed to occur with certain other 5-HT receptor agonists (e.g., 8-hydroxy-*N*,*N*-di-*n*-propylaminotetralin or 8-OH DPAT – now considered a prototypical 5-HT_1A_ receptor agonist). Conversely, DOM^178^ (as well as DOI^179^) failed to substitute in rats trained to discriminate 8-OH DPAT from vehicle. Later, DOI was shown to display low affinity (i.e., *K*_i_ = 19,000 nM) for [^3^H]8-OH DPAT-labeled 5-HT_1A_ receptors.^154^ In contrast, although 5-OMe DMT only resulted in partial generalization (i.e., 50% drug-appropriate responding) in rats trained to discriminate 8-OH DPAT from vehicle,^180^ generalization was seen when the animals were pretreated with a 5-HT_2_ receptor antagonist prior to administration of 5-OMe DMT.^181^ Hence, DOM, unlike 5-OMe DMT, seemed to lack a 5-HT_1A_ serotonin receptor component in its actions. Nevertheless, coadministration of a low dose of 8-OH DPAT (50 μg/kg) in combination with DOM to DOM-trained rats resulted in a leftward shift of the DOM dose–response curve and, when administered in combination with the ED_50_ dose of DOM, resulted in stimulus generalization.^88^ It would appear that although DOM and 8-OH DPAT produce different stimulus effects, and that DOM does not bind at 5-HT_1A_ receptors and 8-OH DPAT does not bind at 5-HT_2_ receptors, 8-OH DPAT is capable of modulating the actions of DOM. Similarly, 8-OH DPAT did not substitute in rats trained to discriminate LSD from vehicle;^182^ however, pretreatment with 8-OH DPAT produced a leftward shift in the LSD dose–response curve.^183^ It has been suggested that 5-HT_1A_ receptor stimulation seems to be a “*non-essential component*” of the LSD stimulus because administration of the training dose of LSD was unaffected by a 5-HT_1A_ receptor antagonist.^183^

Ketanserin (initially referred to as R 41 468) and the structurally related pirenperone were identified as the first 5-HT_2_- versus 5-HT_1_-selective antagonists in the early 1980s,^184,185^ and Colpaert et al.^186^ found that pirenperone was a potent antagonist of LSD in rats trained to discriminate LSD from vehicle. Both ketanserin and pirenperone were soon found to very potently antagonize the stimulus effects of DOM, and DOM-stimulus generalization to, for example, mescaline (**2**), LSD (**8**), and 5-OMe DMT (**9b**), suggesting a common stimulus action via activation of 5-HT_2_ receptors.^187^ Later, the stimulus effect of DOM in monkeys, to which substitution occurred with DOI, *R*(−)DOM, and LSD,^188^ was also blocked with 5-HT_2_ antagonists including ketanserin.^189^ AMI-193 or 8-[3-(4-fluorophenoxy)propyl]-1-phenyl-1,3,8-triazaspiro[4.5]-decanone is a combined 5-HT_2A_/dopamine receptor antagonist with >2,000-fold selectivity over 5-HT_2C_ receptors; this compound potently blocked the stimulus actions of DOM in rats at an antagonist dose of 0.04 mg/kg suggesting that the DOM stimulus is quite likely a 5-HT_2A_- rather than 5-HT_2C_-mediated event.^190^ Fiorella et al.,^191^ using rats trained to discriminate *R*(−)DOM (0.4 mg/kg) from vehicle found that stimulus antagonism was better correlated to the 5-HT_2A_ (r = 0.95) rather than 5-HT_2C_ (r = 0.25) receptor affinity of the 11 antagonists examined.

Aghajanian and colleagues,^192,193^ using electrophysiological techniques to examine various rat brain sites that might mediate the actions of arylalkylamine hallucinogens (including DOI, DOM, DOB, mescaline, and LSD) found that common sites of action were located in the neocortex or in subcortical areas with efferent projections throughout the neocortex and implicated 5-HT_2A_ receptors as playing a role. Activation of these receptors suppressed the firing rate of 5-HT-containing neurons in the midbrain dorsal raphe nucleus.^193^ Although it cannot be assumed that the raphe nucleus is the sole site of action of LSD, using rats trained to discriminate intraperitoneal administration of LSD, stimulus generalization occurred when LSD was administered centrally via stereotaxically implanted indwelling cannulae in the raphe nucleus.^194^ In a pair of rather unique studies, rats trained to discriminate electrical stimulation of the dorsal raphe nucleus generalized to LSD and DOI.^195,196^

With the finding that DOB (**7**) is a high-affinity 5-HT_2A_ receptor agonist, the higher-affinity isomer, *R*(−)DOB, was established as a training drug (training dose = 0.2 mg/kg, i.p.) in rat drug discrimination studies.^197,198^*R*(−)DOB (ED_50_ = 0.05 mg/kg) stimulus generalization occurred with DOM (ED_50_ = 0.24 mg/kg) and LSD (ED_50_ = 0.04 mg/kg) (Figure 5),^198^ and was potently blocked by pirenperone (at 0.03 mg/kg).^179^ Consistent with their affinities for [^3^H]DOB-labeled 5-HT_2A_ receptors (*K*_i_), the order of substitution potency (ED_50_) for several DOB-related agents was: *R*(−)DOB (*K*_i_ = 0.39 nM/ED_50_ = 0.05 mg/kg) > *S*(+)DOB (2.3 nM/0.56 mg/kg) > *N*-monomethyl DOB (7.7 nM/0.82 mg/kg) > *N*,*N*-dimethyl DOB (94 nM/5.36 mg/kg) (Figure 5). The N,N,N-trimethyl quaternary amine analog of DOB (*K*_i_ = 8,250 nM) failed to substitute, due perhaps to its rather low affinity and/or inability to penetrate the blood-brain barrier.^197^ This provided some very useful and applicable *in vitro* and *in vivo* structure–activity and structure-affinity (i.e., SAFIR) information.

**Figure 6:**
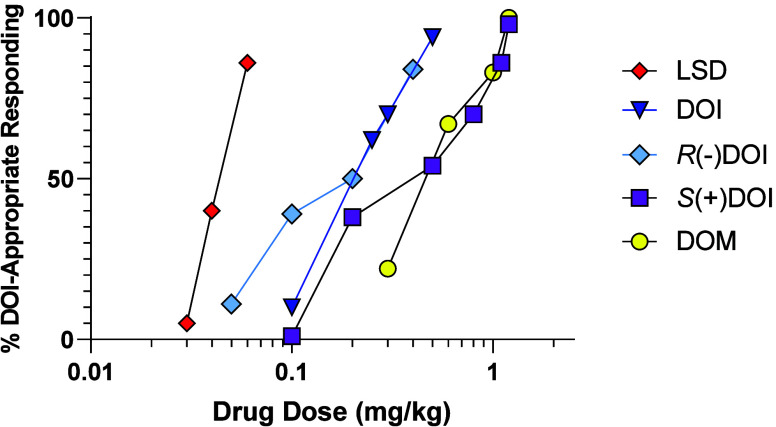
Dose–response curves for several agents in rats trained to discriminate DOI (0.5 mg/kg) from vehicle. The graph was generated from previously published tabular data.^199^

Being another high-affinity agonist at 5-HT_2A_ receptors, DOI also was examined as a training drug in rats (training dose = 0.5 mg/kg, i.p.; ED_50_ = 0.16 mg/kg).^199^ The DOI stimulus was attenuated by pretreatment of the animals with a very low dose (0.05 mg/kg) of the 5-HT_2_ receptor antagonist ketanserin, and DOI stimulus generalization occurred to *R*(−)DOI, *S*(+)DOI, DOM, and LSD (ED_50_ = 0.15, 0.34, 0.49, and 0.05 mg/kg, respectively) (see Figure 6).^199^ Stimulus generalization also occurred with DOB, but not with the 5-HT_1A_ receptor agonist 8-OH DPAT.^179^ Subsequently, others employed DOI (**1**) as a training drug in drug discrimination studies using different training doses and/or schedules of reinforcement. A DOI training dose of 0.63 mg/kg in rats^200^ was blocked by 5-HT_2A_ antagonists, and with a DOI training dose of 0.35 mg/kg, LSD substituted for DOI and the stimulus was completely antagonized by ketanserin (0.5 mg/kg) and cyproheptadine (0.5 mg/kg).^201^ Using a training dose of 0.75 mg/kg, Smith et al.^202^ found that chronic administration of DOI resulted in behavioral tolerance and suggested on the basis of autoradiographic studies, that neuroadaptive changes in 5-HT_2A_, not 5-HT_2C_, receptors accounted for its stimulus effect. Marona-Lewicka et al.^204^ reported that unlike an LSD stimulus that involves a role for 5-HT_2_ and dopamine D_4_ receptors, the DOI stimulus does not involve a dopaminergic component. Overall, the various results were in agreement with the conclusion that DOI serves as an effective discriminative stimulus, that DOI-stimulus generalization can occur with certain other arylalkylamine hallucinogens, and that the stimulus is 5-HT_2_-receptor (likely 5-HT_2A_ rather than 5-HT_2C_) mediated. However, far fewer agents have been examined in DOB- or DOI-trained animals than in DOM-trained animals; nevertheless, using either DOM, *R*(−)DOB, or DOI as training drug in drug discrimination studies, the results were quite consistent in that a 5-HT_2A_ receptor agonist mechanism primarily underlies their *common* stimulus effects.

We found for a series of serotonergic hallucinogens that their human potency was correlated with their stimulus generalization potency in DOM-trained animals (reviewed^62^). For 20 such agents (including, for example, DOI, DOM, DOET, DOPR, and 2,4,5-TMA) that their 5-HT_2A_ receptor affinity was also significantly correlated (r = 0.94) with their human hallucinogenic potency (reviewed^70,89^). Interestingly, there was also a correlation between rat 5-HT_2A_ and 5-HT_2C_ (termed 5-HT_1C_ at the time) receptor affinity for DOX-type agents.^133^ Later, 5-HT_2A_ and 5-HT_2C_ receptor affinity was found to be less robustly correlated (r = 0.66), but achieved significance (r = 0.89) if two outliers [i.e., 1-(2,4-dimethoxyphenyl)-2-aminopropane and 1(2,4-dimethoxy-5-ethoxyphenyl)-2-aminopropane] were removed.^70^ Perhaps, then, correlations between behavioral actions and 5-HT_2*A*/2C_ receptor affinity are not surprising given that their 5-HT_2A_ and 5-HT_2C_ receptor affinity are intercorrelated.^134^ More recently, Luethi and Liechti^203^ demonstrated a significant Spearman rank-order correlation between the human hallucinogenic potency of 35 agents both with 5-HT_2A_ and 5-HT_2C_ receptor affinity even though there was only a single agent (i.e., mescaline) common to the two^70,203^ investigations. Other findings of the latter study was that there was no correlation between human data and 5-HT_1A_ or 5-HT_2B_ receptor affinity, that there was a significant intercorrelation between 5-HT_2A_ and 5-HT_2C_ receptor affinity, and that although all agents were 5-HT_2A_ agonists, there was no correlation between their human potency and agonist action as measured using a calcium mobilization assay.^203^

## DOI Analogues

Removal or introduction of substituents to the DOX structure has not only provided structure–activity data but has also resulted in a number of useful agents. To illustrate the trend, analogs of DOM and DOB will be described for comparison followed by analogs of DOI.

### α-Desmethyl Analogs

**Figure 7:**
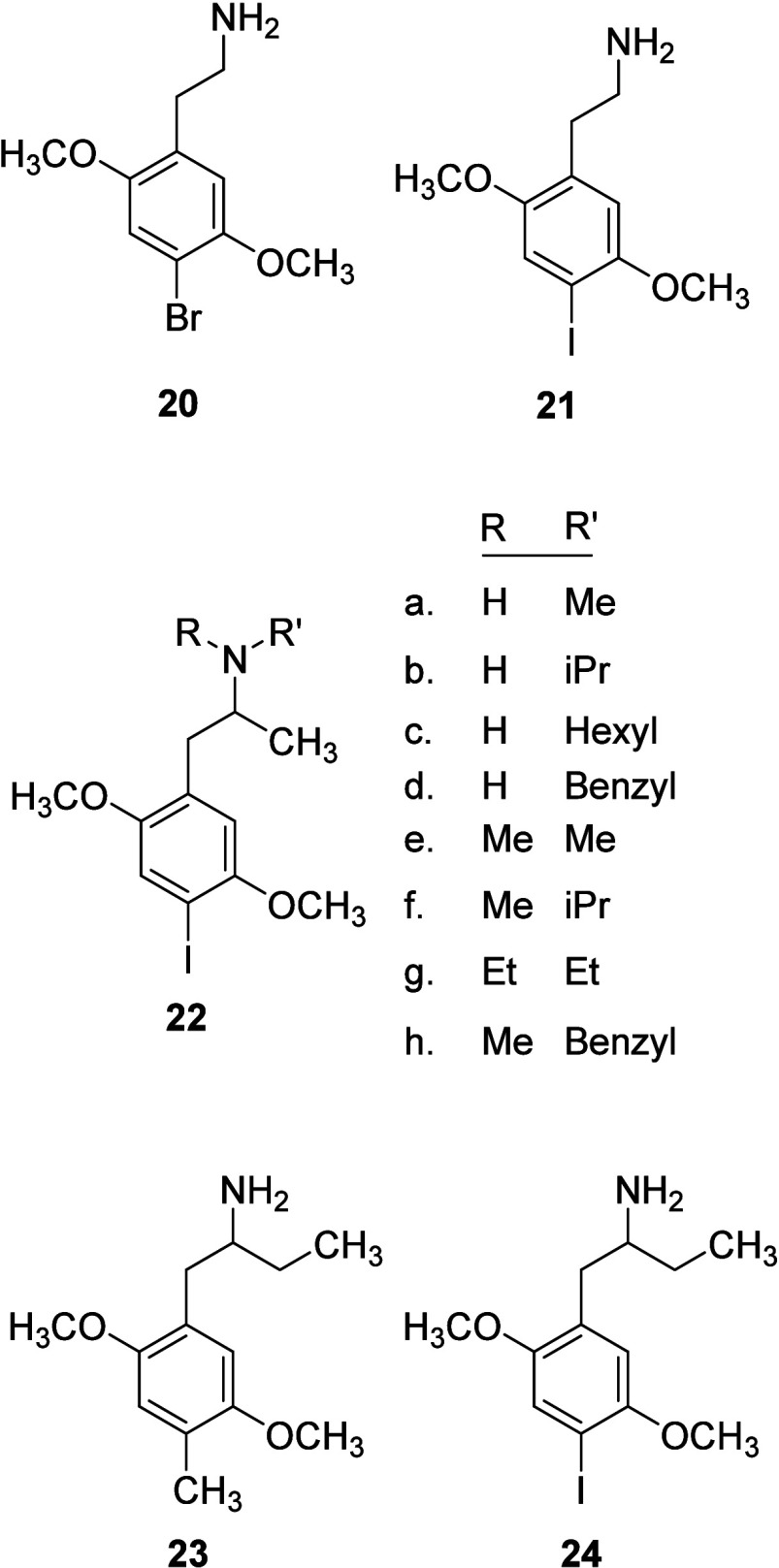
DOX-related agents described in the text.

Removal of the α-methyl group of phenylisopropylamine DOX-related agents typically results in a slight (usually <10-fold) decrease in *in vivo* potency but in little change in 5-HT_2_ receptor affinity. An explanation for this is that the α-desmethyl analogues are less readily able to penetrate the blood-brain barrier and/or are more quickly metabolized in vivo. The α-desmethyl counterparts of DOX-related agents are commonly referred to as 2C-X compounds. That is, the 3-atom carbon chain of phenylisopropylamines such as DOX is shortened to a 2-atom phenylethylamine moiety (hence, the “2C”, with X representing the 4-position substituent as it does with DOX agents); alternatively, these agents might be viewed as DOX compounds where the α-methyl group has been replaced by a hydrogen atom. α-Desmethyl DOB (or 2C–B, **20**; Figure 7) binds at 5-HT_2A_ receptors (*K*_i_ = 32 nM and 1.0 nM at [^3^H]ketanserin-labeled and [^3^H]DOB-labeled receptors, respectively), and DOB (*K*_i_ = 40 nM and 0.79 nM), binds with comparable affinity. In drug discrimination studies using rats, α-desmethyl DOB substituted in DOM-trained and *R*(−)DOB-trained animals with potencies (ED_50_ values) of 0.67 and 0.48 mg/kg; in the latter group of animals, the potencies (ED_50_ values) of DOM and *R*(−)DOB were 0.24 and 0.05 mg/kg, respectively.^197,205^ Likewise, α-desmethyl DOM (also known as 2C-M) (ED_50_ = 1.31 mg/kg) substituted in DOM-trained rats (DOM ED_50_ = 0.44 mg/kg) and was four times less potent than DOM;^84^ its affinity for [^3^H]ketanserin-labeled rat cortical 5-HT_2_ receptors was similar to that of DOM (*K*_i_ = 110 nM and 100 nM, respectively) but less than the affinity of *R*(−)DOI (*K*_i_ = 9.9 nM).^141^ An advantage of studying α-desmethyl analogues is that they lack a chiral center and avoid the synthesis and evaluation of the individual optical isomers.

α-Desmethyl DOI or 2C–I (**21**) behaved in like manner to its α-desmethyl DOM and DOB counterparts. An early QSAR study found that α-desmethyl DOB and α-desmethyl DOI should be the most potent in an examined series of hallucinogenic agents in human studies;^49,206^ the QSAR study was performed prior to obtaining human data on α-desmethyl DOI. However, this was found to be the case once human data with α-desmethyl DOI were obtained.^206,207^ α-Desmethyl DOI binds at [^125^I]DOI-labeled 5-HT_2A_ receptors with high affinity (*K*_i_ = 0.62 nM),^208^ and [^125^I]α-desmethyl DOI has been demonstrated to label 5-HT_2A_ receptors.^209^ The [^131^I]-radiolabeled version also has been used in animal imaging studies.^210^ α-Desmethyl DOI substituted for LSD in drug discrimination studies and produced the HTR in mice; its potency relative to other agents in these assays has been compared.^126^ Moya et al.^211^ found, upon comparison of the HTR of a series of 4-substituted DOX analogs bearing or lacking an α-methyl group, that subtle changes in ligand structure can result in significant differences in the cellular (arachidonic acid release and inositol phosphate accumulation) signaling profile, and that the presence or absence of an α-methyl group (e.g., of DOI, DOM, DOB) influences their efficacy at 5-HT_2A_ (and 5-HT_2C_) receptors.^212^

### Simple N-Alkyl Analogues

Introduction of simple terminal-amine substituents such as an N-monomethyl, N,N-dimethyl, or a related short alkyl group to phenylalkylamine DOX agonists results in a small decrease in their 5-HT_2A_ receptor affinity and human (where data are available) and animal behavioral potency that progressively worsens as the alkyl group is extended in length. This was described above, for example, for N-substituted analogs of DOB;^197^ N-monomethylation of DOM or *R*(−)DOM has resulted in reduced 5-HT_2A_ receptor affinity (*K*_i_ = 415 nM and 260 nM, respectively, relative to 100 nM for DOM),^141^ and in about a 10-fold reduction in substitution potency in drug discrimination studies with DOM-trained rats.^84,87^ N,N-Dimethylation of α-desmethyl DOM (i.e., *N,N*-dimethyl 2C-M) (ED_50_ = 5.37 mg/kg) resulted in a 4-fold decease in potency in these same animals.^84^ Various N-monosubstituted and N,N-disubstituted analogs of DOI have been described (e.g., **22a**-**22h**; Figure 7),^15^ but pharmacological data are minimal or nonexistent. Human data have been reported for the N,N-dimethyl analogue **22e**, which has been referred to as IDNNA (see below). The ^11^C N-monomethyl and N,N-dimethyl analogues of DOI have been prepared and examined as PET imaging agents.^213^

### α-Ethyl Homologues

Standridge et al.^55,214^ in a “*program directed at potential non-hallucinogenic performance-enhancing agents*” first synthesized and examined the α-ethyl homologue of DOM (i.e., BL-3912; **23**) and its optical isomers, followed by an investigation of a series of DOX-related agents. The latter study included the α-ethyl homologues of DOM, DOET, DOPR and, in some cases, their optical isomers; the α-ethyl homologue of *R*(−)DOI was also prepared and examined.^214^ Some preliminary animal pharmacological data were provided revealing that most of the analogs possess a “*low hallucinogenic potential*” relative to DOM. The ^131^I-substituted compound (i.e., the radioiodinated analogue of the 4-iodo counterpart of BL-3912, or the α-ethyl homologue of DOI, has been reported.^210^

The α-ethyl homologue of DOM, **23**, substituted in LSD-trained rats.^215^ Confirming its stimulus actions, in DOM-trained rats the α-ethyl homologue of DOM and its *R*(−)-isomer substituted for DOM.^84^ The corresponding DOI homologue **24** was not investigated in these studies. A more recent study examined both the α-ethyl (i.e., **24**) and α-propyl homologues of DOI (and other compounds) in various assays including activation of 5-HT_2A_-mediated second messenger systems.^216^ Because these α-ethyl compounds reportedly lack human hallucinogenic action (where this was investigated; see below), a compelling argument was made that they might be effective for the treatment of certain neuropsychiatric disorders without producing the undesired psychoactive actions of their hallucinogenic DOX counterparts.^216^

### N-Benzyl Analogs

Sixty years ago, Ariëns and colleagues^217,218^ found that introduction of lengthy/bulky substituents to the terminal amine of certain agonists converted them to antagonists. Today, it is thought that these added substituents might interact within the orthosteric binding site of a target protein but utilize a receptor feature not required by agonists. Hence, we endeavored to use this concept to develop novel 5-HT_2A_ receptor antagonists; that is, will adding the appropriate terminal amine substituent to DOX-type agonist agents offer a new approach to the development of novel 5-HT_2A_ receptor antagonists? We began this study with simple N-alkyl substituents (see above discussion with simple N-alkyl groups) and then increased the bulk and length of the substituent. What we found was that certain lengthy/bulky amine substituents (to a degree) enhanced the affinity of α-desmethyl DOB. The study began with small N-monoalkyl groups (e.g., methyl, *n*-propyl) that were then extended to longer or more bulky groups such as the N-phenylethyl analogue **25** and longer substituents. Interestingly, it was found that N-benzyl analogs of phenylalkylamines (as well as that of indolealkylamines–compounds that will not be discussed here) actually resulted in enhanced affinity.^219^ N-Benzyl analogs of α-desmethyl DOB (e.g., **26**) displayed significantly enhanced affinity for 5-HT_2A_ receptors (and selectivity for 5-HT_2A_ over 5-HT_2C_ receptors), and an affinity higher than their longer-chain analogs.^219^ Because they displayed higher affinity for agonist-labeled versus antagonist-labeled 5-HT_2A_ receptors (see Table 4 for some representative data), we speculated that they would behave as agonists. That is, 5-HT_2A_ receptor agonists typically display higher affinity for agonist-labeled 5-HT_2A_ receptors than they do for radiolabeled antagonist-labeled receptors. These studies were never followed up by us. However, others have examined tritiated versions of N-benzyl **21**.^220^

**Table 4:**
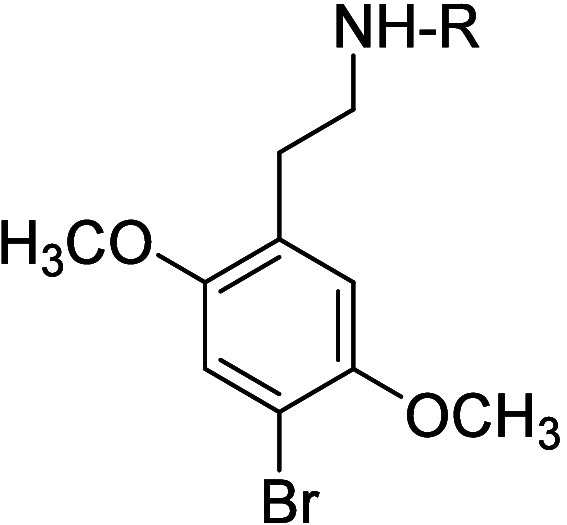
Affinities of Selected N-Alkyl Analogs of **11** at [^3^H]Ketanserin- and [^125^I]DOI-Labeled 5-HT_2A_ Receptors^219^

R	[^3^H]Ketanserin *K*_i_ (nM)	[^125^I]DOI *K*_i_ (nM)
-H	34	1.0
-CH_2_Ph (**26**)	16	0.3
-(CH_2_)_2_Ph (**25**)	83	23
-(CH_2_)_3_Ph	-	23
-(CH_2_)_4_Ph	-	27
-(CH_2_)_2_Ph(4′-I)	22	0.4

In a very productive series of studies, Nichols and colleagues prepared and examined a number of conformationally constrained DOX-related analogs. Whereas conformationally constrained compound **27** displayed low affinity for 5-HT_2A_ receptors, compound **28** acted as an agonist.^221^ Constraining both methoxy substituents of DOX analogs as dihydrofurans (i.e., **29**) was found to be favorable. For example, **29** where X = Br displayed high affinity for [^3^H]ketanserin- and [^125^I]DOI-labeled 5-HT_2A_ receptors (*K*_i_ = 10.7 and 0.48 nM, respectively).^221,222^ This compound (ED_50_ = 0.06 mg/kg) was nearly equipotent with LSD (ED_50_ = 0.04 mg/kg) in rats trained to discriminate LSD from vehicle.

**Figure 8:**
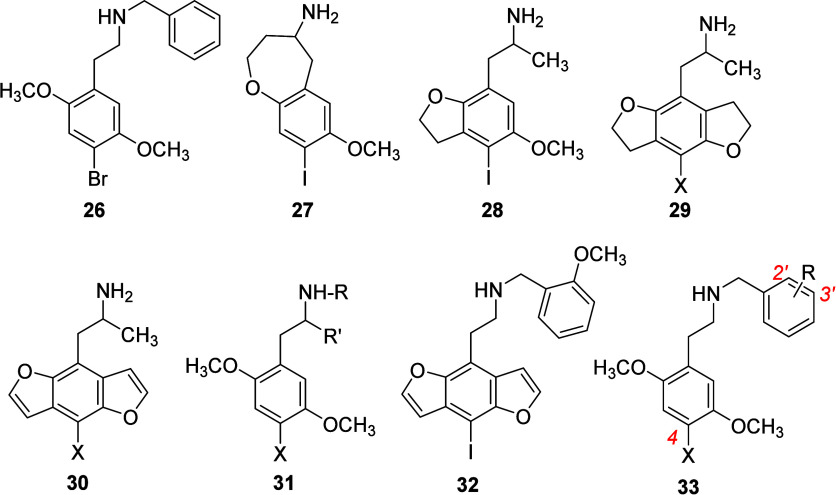
Chemical structures of compounds **26**-**33**.

Subsequent studies examined the optical isomers of a series of **29** analogs, their fully aromatized counterparts **30**, and their individual optical isomers.^223^ For example, **30** where X = I acted as an agonist with the *R*-isomer (**30**, X = I; *K*_i_ values for [^3^H]DOB- and [^125^I]DOI-labeled receptors of 0.31 and 0.11 nM, respectively) binding with slightly higher affinity than its *S*-enantiomer (*K*_i_ = 0.68 and 0.25 nM, respectively). Continued effort led to a series of N-substituted analogs **31** (where R′ = H or −CH_3_, and X was varied). For example, **32** (25I-NBOMe; *K*_i_ = 0.087 nM at [^3^H]DOB-labeled 5-HT_2A_ receptors), behaved as an agonist and displayed higher affinity than 2C–I (**12**; *K*_i_ = 0.62 nM), substituted in LSD-trained rats, and was very potent in producing the mouse HTR.^126^ These compounds represent some of the highest affinity 5-HT_2A_ agonists yet reported.^208,222^ In studies focused on developing an agent for positron-emission imaging, Hansen et al.^224^ examined 48 α-desmethyl analogues of 2,5-DMA that varied in substituents at the 2′- and 3′-positions of the N-benzyl group and possessed a dozen different aryl 4-position substituents (i.e., **33**, Figure 8) with respect to their h5-HT_2A_ versus r5-HT_2C_ receptor affinity (using [^3^H]ketanserin and [^3^H]mesulergine, respectively, as radioligands) and functional activity as determined using an inositol phosphate turnover assay. Compound **33** where X = Et and R = 2′–OH displayed the highest 5-HT_2A_ receptor affinity (*K*_i_ = 0.29 nM) whereas iodo compound **33** (where X = I and R = 2′–OH) was the most potent (EC_50_ = 0.074 nM) and 400-fold 5-HT_2A_-selective in the functional assay.^224^

## Human Studies

Apart from anecdotal reports that lack citable documentation and, most frequently, lack authentication as to the exact identity of what was being evaluated, relatively little reliable human data have been reported on DOI or its analogues. Some of the most cited and comparative human data have been described by Shulgin and co-workers;^15,21,225^ the identity of the agents was rigorously established and other parameters provided (e.g., dose, route of administration, time of onset, duration of action). Unfortunately, there is often little information on the number of subjects in which an agent was examined. DOI was evaluated at various oral doses and found to be psychoactive at 1.5–3.0 mg with an extended (16–30 h) duration of action. *R*(−)DOI (at 1.0 and 2.3 mg) was found to be more potent than the *S*(+)-isomer (examined at 6.3 mg). Hence, the human findings with the DOI isomers are consistent with the results of radioligand binding studies and various *in vivo* conditioned and unconditioned animal behaviors (*vide supra*) in that the isomers produce a stereoselective, not stereospecific, effect. For purpose of comparison, the potency range for its 4-methyl counterpart, DOM, is stated to be 3–10 mg.^21^ Data for several other DOX analogs can be found in Table 1 for comparison.

Removal of the α-methyl group of DOX-related agents reduces human potency by “*sometimes up to an order of magnitude*”.^207^ α-Desmethyl DOM (or 2C-M) is active in humans with an oral dose range of 20–60 mg;^21^ that is, it is approximately six to seven times less potent than DOM. Likewise, α-desmethyl DOB is about ten times less potent than DOB.^21^ α-Desmethyl DOI (i.e., **21**) was found to be approximately eight times less potent (dose range = 14–22 mg, p.o.) and of shorter duration (6–12 h) than DOI itself.^21,49,207^ The N,N-dimethyl analogue of DOI (or IDNNA, **22e**) showed “*no activity*” at a dose where DOI is active (i.e., at 2.6 mg, p.o), and the α-ethyl homologue of DOI (i.e., **24**) was without central effect at oral doses of up to 4.0 mg.^15,21^

Due perhaps to their very high affinity as 5-HT_2A_ receptor agonists, a dose of 50 μg of **32** being sufficient to produce psychoactive effects, abuse of **32** and several related agents has led to toxicity problems including death.^226^ See also Pelletier et al.^227^ for a recent summary.

In general, it would be gratifying if data from animal behavioral studies were in agreement with human findings, and this is very often the case. For example, the results of human studies are generally consistent with the above-mentioned drug discrimination and HTR data in that DOI is more potent than DOM, the *R*-isomers of DOX-type agents are more potent than their *S*-enantiomers (with action being stereoselective rather than stereospecific), deletion of the α-methyl group of DOX agents results in somewhat diminished potency, and N-alkylation (except for the N-benzyl analogs described above) decreases or abolishes activity. However, it should be mentioned that exceptions exist. Whereas BL-3912 (**23**) substituted in LSD-trained animals^215^ and both BL-3912 and its *R*(−)isomer substituted in DOM-trained rats^84^ – suggesting that BL-3912 might represent a hallucinogenic agent–human results, although lacking detail–do not seem to support this. Moreover, BL-3912 was active in the HTR assay, with the *R*-isomer being more potent than the *S*-isomer (although the response was “*markedly attenuated*” compared to active control).^216^ In humans, oral doses of the *R*(−)isomer of **23** “*increased mental alertness*” at 25–50 mg, and 50–100 mg produced “*relief of manic depression*”.^15^ Doses of **23** up to 270 mg in normal human subjects produced “*euphoria and other LSD-like effects but neither perceptual changes nor hallucinations*”.^215^ To put this in perspective, the parent structure, DOM, is active in human subjects at doses of 3–10 mg.^21^

## Other Studies Utilizing DOI

Although by no means comprehensive, Table 5 provides representative examples of other types of studies that have been conducted with DOI; admittedly, hundreds more studies could have been cited.^16^ Early studies simply examined its *in vitro* and *in vivo* pharmacology whereas later studies, once it was realized that DOI is a 5-HT_2_ receptor agonist, utilized DOI or a radiolabeled version thereof to implicate or eliminate a role for 5-HT_2_ receptor involvement in a specific action. In many early cases, the effects of DOI were blocked by various nonselective serotonin antagonists and, later, by more selective 5-HT_2_ antagonists such as ketanserin and pirenperone following their discovery. The breadth, scope, and applicational utility of DOI in various fields of investigation are exemplified.

**Table 5:** Some Representative Studies Utilizing DOI^a^

Topic	Reference
Anorectic actions	(228−232)
Anxiety, depression, schizophrenia	(233−246)
Auditory filtering	(247)
Blood pressure and heart rate	(248−250)
Body shakes and head-bobs in rabbits	(251−253)
Brain-derived neurotrophic factor (BDNF) regulation	(254−258)
Circadian rhythm	(259)
Cocaine (rat hyperactivity, self-administration)	(260, 261)
Compulsive behavior (mice)	(262)
Conditioned taste aversion in rats	(263)
Corticosterone and rodent behavior	(264, 265)
Cyclic AMP response element binding protein (CREB)	(266)
Dorsal raphe neuronal firing inhibited in rat	(267)
Ethanol consumption by mice/rats	(101, 268)
Forepaw treading by rats	(269)
Glutamate–involvement in DOI actions	(270, 271)
Head-twitch response, tonic activation	(272)
Hyperglycemia in rats	(273)
Hyperthermia in rats	(264, 274−277)
Inflammation	(278, 279)
Intracranial self-stimulation (ICSS)	(280)
Intraocular pressure (glaucoma)	(281)
Locomotor activity in rodents	(282−286)
Malignant hyperthermia	(287−289)
PI Turnover	(271, 290, 291)
Platelet aggregation inhibition	(292−295)
Prolactin secretion in rodents	(296, 297)
Self-administration (lack of by Rhesus monkeys)	(298)
Serotonin syndrome in rats	(299)
Sexual performance of male and female rats	(300−305)
Signaling (DOI versus lisuride)	(306)
Spinal 5-HT_2_ receptors involved in rat motor behavior and nociception	(307−309)
Visual sensorimotor gating	(310)
5-HT_2_ receptor down-regulation	(311, 312)

a A number of the studies mentioned here have seen follow-up or related investigations that are not necessarily cited.

Because serotonergic psychedelics are currently receiving renewed interest as potential psychotherapeutic agents it is, perhaps, logical that phenylalkylamines such as DOI be considered. With respect to DOI, very low doses (0.1 mg/kg) showed an antidepressant-like effect in animals (e.g., mouse forced-swim and tail-suspension tests) without inducing the HTR, and a role for 5-HT_2A_ receptors was implicated.^313^ However, there has been some controversy regarding the use of serotonergic psychedelic agents for treatment-resistant depression and anxiety and whether or not this beneficial therapeutic effect is inextricably linked to their attendant hallucinogenic actions (for example, see: Jaster and González-Maeso,^136^ Takaba et al.,^313^ Lewis et al.,^314^ Cameron et al.,^315^ and Wallach et al.^316^ for extended discussions). Nearly 40 years ago, it was speculated that if such agents act via a 5-HT_2_ agonist mechanism, “*appropriate structural modifications could give rise to 5-HT*_2_*agonists that are not hallucinogenic. In other words, 5-HT*_2_*agonist action might simply be a predisposing factor for such activity; hallucinogens might be 5-HT*_2_*agonists, but will all 5-HT*_2_*agonists be hallucinogenic?*”.^45^ Recent studies^216,314,317,318^ now have identified several 5-HT_2_ agonists that seemingly lack the human hallucinogenic actions, or hallucinogen-like actions in animals, associated with serotonergic psychedelic agents. by virtue of their different effects involving biased signaling, synaptic plasticity, agonism versus partial agonism, and possibly their specific brain-site(s) of action.^e.g.^^4,114,314−316,318−320^

## Conclusion

It has been 50 years since its inauspicious beginnings as part of a DOM-related structure–activity study on hallucinogenic agents by Coutts and Malicky,^32^ but regardless of whether or not DOI achieves clinical ascendency, it deserves acclaim for the considerable role it has played in understanding serotonin receptor pharmacology and function. Being among one of the first recognized 5-HT_2_ receptor agonists (although with little selectivity among the three 5-HT_2_ receptor subfamilies), DOI and DOI analogues assisted, together with antagonists such as ketanserin, pirenperone, and other antagonists since developed, in unraveling some of the mysteries of 5-HT receptor subtypes. DOI did so, in part, by being recognized as a 5-HT_2_ receptor agonist, contributing to formulation of structure–activity (and QSAR) relationships, leading to the development novel agonist radioligands for binding and imaging studies, aiding animal behavioral studies (e.g., drug discrimination, the HTR) to classify serotonergic psychedelic and other agents, assisting the identification of novel 5-HT_2_ receptor antagonists, and the introduction of some of the most potent 5-HT_2_ receptor agonists reported to date. It has been, and continues to be, especially valuable in achieving and extending the current state-of-affairs in 5-HT receptor research and contributing as a useful tool toward the goal of developing novel and safe psychotherapeutic agents. Nevertheless, future studies using this agent might be hampered somewhat because DOI is now being considered for control as a Schedule I substance by the U.S. DEA.^321^

## Notes

No new data are provided here. All data have been previously reported and are cited.

## Abbreviations

AMI-193: 8-[3-(4-fluorophenoxy)propyl]-1-phenyl-1,3,8-triazaspiro[4.5]-decanone
DEA: Drug Enforcement Administration
DMT: *N,N*-dimethyltryptamine
DOB: 1-(4-bromo-2,5-dimethoxyphenyl)-2-aminopropane
DOC: 1-(4-chloro-2,5-dimethoxyphenyl)-2-aminopropane
DOET: 1-(2,5-dimethoxy-4-ethyl-phenyl)-2-aminopropane
DOF: 1-(2,5-dimethoxy-4-fluorophenyl)-2-aminopropane)
DOI: 1-(2,5-dimethoxy-4-iodophenyl)-2-aminopropane
DOM: 1-(2,5-dimethoxy-4-methyl-phenyl)-2-aminopropane
DOPP: 1-[2,5-dimethoxy-4-(n-3-phenylpropy)phenyl)-2-aminopropane
DOPR: 1-(2,5-dimethoxy-4-*n*-propylphenyl)-2-aminopropane
DOX: 1-(2,5-dimethoxy-4-X-phenyl)-2-aminopropane (a general structure where the X substituent is varied)
ESR: ear-scratch response
HTR: head-twitch response
IDNNA: the N,N-dimethyl analogue of DOM
LSD: (+)lysergic acid diethylamide
PET: positron emission tomography
QSAR: quantitative structure–activity relationships
SPECT: single-photon emission computed tomography
TRD: treatment-resistant depression
2C–B: α-desmethyl DOB
2C–I: α-desmethyl DOI
2C-M: α-desmethyl DOM
2,5-DMA: 1-(2,5-dimethoxyphenyl)-2-aminopropane
4-OMe DMT: 4-methoxy-*N*,*N*-dimethyltryptamine
5-HT: 5-hydroxytrptamine (serotonin)
5-OMe DMT: 5-methoxy-*N*,*N*-dimethyltryptamine
6-OMe DMT: 6-methoxy-*N*,*N*-dimethylytryptamine
8-OH DPAT: 8-hydroxy-2-(di-*n*-propylamino)tetralin

## References

[ref1] Geyer M. A. A brief historical overview of psychedelic research. Biol. Psychiatry Cogn. Neurosci. Neuroimaging 2023, 23, S2451-9022(23)00314-2 10.1016/j.bpsc.2023.11.003. 38000715

[ref2] Holze F.; Singh N.; Liechti M. E.; D’Souza D. C. Serotonergic psychedelics - a comparative review comparing the efficacy, safety, pharmacokinetics and binding profile of serotonergic psychedelics. Biol. Psychiatry Cogn. Neurosci. Neuroimaging 2024, 30, S2451-9022(24)00020-X 10.1016/j.bpsc.2024.01.007. 38301886

[ref3] Kwan A. C.; Olson D. E.; Preller K. H.; Roth B. L. The neural basis of psychedelic action. Nature Neurosci. 2022, 25, 1407–1419. 10.1038/s41593-022-01177-4. 36280799 PMC9641582

[ref4] Gumpper R. H.; Roth B. L. Psychedelics: preclinical insights provide directions for future research. Neuropsychopharmacology 2024, 49, 119–112. 10.1038/s41386-023-01567-7. 36932180 PMC10700551

[ref5] Roth B. L.; Gumpper R. H. Psychedelics as transformative therapeutics. Am. J. Psychiat. 2023, 180, 340–347. 10.1176/appi.ajp.20230172. 37122272

[ref6] Calder A. E.; Hasler G. Towards an understanding of psychedelic-induced neuroplasticity. Neuropsychopharmacology 2023, 48, 104–112. 10.1038/s41386-022-01389-z. 36123427 PMC9700802

[ref7] Chiu Y. T.; Deutch A. Y.; Wang W.; Schmitz G. P.; Huang K. L.; Kocak D. D.; Llorach P.; Bowyer K.; Liu B.; Sciaky N.; Hua K.; Chen C.; Mott S. E.; Niehaus J.; DiBerto J. F.; English J.; Walsh J. J.; Scherrer G.; Herman M. A.; Wu Z.; Wetsel W. C.; Roth B. L. A suite of engineered mice for interrogating psychedelic drug actions. bioRxiv [Preprint]. 2023, Sep 26:2023.09.25.559347. 10.1101/2023.09.25.559347. PMID: 37808655; PMCID: PMC10557740. PMC13533840 42563032

[ref8] Blake A.; Fordyce B. A.; Roth B. L. Making sense of psychedelics in the CNS. Int. J. Neuropsychopharmacol. 2024, in press. 10.1093/ijnp/pyae007. PMC10888522 38289825

[ref9] van den Berg M.; Magaraggia I.; Schreiber R.; Hillhouse T. M.; Porter J. H. How to account for hallucinations in the interpretation of the antidepressant effects of psychedelics: a translational framework. Psychopharmacology (Berl.) 2022, 239, 1853–1879. 10.1007/s00213-022-06106-8. 35348806 PMC9166823

[ref10] Yao H.; Wang X.; Chi J.; Chen H.; Liu Y.; Yang J.; Yu J.; Ruan Y.; Xiang X.; Pi J.; et al. Exploring novel antidepressants targeting G protein-coupled receptors and key membrane receptors based on molecular structures. Molecules 2024, 29, 964–1003. 10.3390/molecules29050964. 38474476 PMC10934989

[ref11] Glennon R. A.; Iversen L. Antidepressants In: Burger’s Medicinal Chemistry and Drug Discovery, Vol. 8, CNS Agents. Ed. Glennon R. A. Eighth ed., Series Ed. Abraham D. J.; Myers M. R.; Wiley: NY, 2021; pp 331–386.

[ref12] Psychedelic Drugs: Considerations for Clinical Investigations; U.S. Food and Drug Administration. https://www.fda.gov/media/169694/download (accessed January 1, 2024).

[ref13] Glennon R. A. The 2014 Philip S. Portoghese Medicinal Chemistry Lectureship: The ″Phenylalkylaminome″ with a focus on selected drugs of abuse. J. Med. Chem. 2017, 60, 2605–2628. 10.1021/acs.jmedchem.7b00085. 28244748 PMC5824997

[ref14] Kaiho T. Pharmaceuticals: therapeutic agents. Chapter 23. In: Iodine Chemistry and Applications; Kaiho T., Ed.; John Wiley and Sons: New York, 2014; pp 433–437. 10.1002/9781118909911.

[ref15] Shulgin A. T.; Manning T.; Daley P. F. The Shulgin Index: Psychedelic Phenethylamines and Related Compounds. Transform Press: Berkeley. CA, 2011.

[ref16] PubMed search for “1-(2,5-dimethoxy-4-iodophenyl)-2-aminopropane” or “2,5-dimethoxy-4-iodoamphetamine”, revealed 815 and 820 publications, respectively (accessed February 14, 2024).

[ref17] Behavioral Neurobiology of Psychedelic Drugs; Halberstadt A. L., Vollenweider F. X., Nichols D. E., Eds.; Springer: Berlin, Heidelberg, 2018. 10.1007/978-3-662-55880-5.

[ref18] Glennon R. A.; Dukat M. Quipazine: classical hallucinogen? novel psychedelic?. Aust. J. Chem. 2023, 76, 288–298. 10.1071/CH22256.

[ref19] Schifano F.; Napoletano F.; Chiappini S.; Orsolini L.; Guirguis A.; Corkery J. M.; Bonaccorso S.; Ricciardi A.; Scherbaum N.; Vento A. New psychoactive substances (NPS), psychedelic experiences and dissociation: clinical and clinical pharmacological issues. Cur. Addict, Rep. 2019, 6, 140–152. 10.1007/s40429-019-00249-z.

[ref20] Fordyce B. A.; Roth B. L. Making sense of psychedelics in the CNS. Int. J. Neuropsychopharmacol. 2024, 27, pyae007 10.1093/ijnp/pyae007. 38289825 PMC10888522

[ref21] Shulgin A.; Shulgin A. PIHKAL; Joy D., Ed.; Transform Press: Berkeley, CA. 1991.

[ref22] Trout K.; Daley P. F. The origin of 2,5-dimethoxy-4-methylamphetamine (DOM, STP). Drug Test. Anal. 2024, 10.1002/dta.3667. 38419183

[ref23] Snyder S. H.; Faillace L.; Hollister L. 2,5-Dimethoxy-4-methyl-amphetamine (STP): a new hallucinogenic drug. Science 1967, 158, 669–670. 10.1126/science.158.3801.669. 4860952

[ref24] Snyder S. H.; Faillace L. A.; Weingartner H. DOM STP, a new hallucinogenic drug, and DOET: effects in normal subjects. Am. J. Psychiat. 1968, 125, 357–364. 10.1176/ajp.125.3.357. 4385937

[ref25] Snyder S. H.; Weingartner H.; Faillace L. A. DOET (2,5-dimethoxy-4-ethylamphetamine), a new psychotropic drug. Effects of varying doses in man. Arch. Gen. Psychiat. 1971, 24, 50–55. 10.1001/archpsyc.1971.01750070052006. 4923215

[ref26] Snyder S. H.; Weingartner H.; Faillace L. A. DOET (2,5-Dimethoxy-4-ethylamphetamine) and DOM (STP) (2,5-dimethoxy-4-methylamphetamine), new psychotropic agents: Their effects in man. In: Psychedelics: The Uses and Implications of Hallucinogenic Drugs; Aaronson B., Osmond H., Eds.; Schenkman Publishing Co.: Cambridge, MA,1971; pp, 247–264.

[ref27] Hollister L. E.; Macnicol M. F.; Gillespie H. K. An hallucinogenic amphetamine analog (DOM) in man. Psychopharmacologia 1969, 14, 62–73. 10.1007/BF00401535. 5351858

[ref28] Shulgin A. T.; Sargent T.; Naranjo C. Structure--activity relationships of one-ring psychotomimetics. Nature 1969, 221, 537–541. 10.1038/221537a0. 5789297

[ref29] Snyder S. H.; Faillace L. A.; Weingartner H. A new psychotropic agent. Arch. Gen. Psychiat. 1969, 21, 95–101. 10.1001/archpsyc.1969.01740190097014. 4389442

[ref30] Shulgin A. T. 4-Alkyl-dialkoxy-α-methylphenethylamines and their pharmacologically acceptable salts. U.S. Patent US3,547,999. Dec. 15, 1970.

[ref31] Nichols D. E.; Barfknecht C. F.; Rusterholz D. B.; Benington F.; Morin R. D. Asymmetric synthesis of psychotomimetic phenylisopropylamines. J. Med. Chem. 1973, 16, 480–483. 10.1021/jm00263a013. 4718460

[ref32] Coutts R. T.; Malicky J. L. The synthesis of some analogs of the hallucinogen 1-(2,5-dimethoxy-4-methylphenyl)-2-aminopropane (DOM). Can. J. Chem. 1973, 51, 1402–1409. 10.1139/v73-210.

[ref33] Glennon R. A.; Young R.; Hauck A. E. Structure-activity studies on methoxy-substituted phenylisopropylamines using drug discrimination methodology. Pharmacol., Biochem. Behav. 1985, 22, 723–729. 10.1016/0091-3057(85)90520-9. 3839309

[ref34] Quinteros-Muñoz D.; Sáez-Briones P.; Díaz-Véliz G.; Mora-Gutiérrez S.; Rebolledo-Fuentes M.; Cassels B. K. Behavioral profiles in rats distinguish among ″ecstasy,″ methamphetamine and 2,5-dimethoxy-4-iodoamphetamine: Mixed effects for ″ecstasy″ analogues. Behav. Neurosci. 2010, 124, 662–676. 10.1037/a0020827. 20939666

[ref35] Snyder S. H. Catecholamines in the brain as mediators of amphetamine psychosis. Arch. Gen. Psychiatry 1972, 27, 169–179. 10.1001/archpsyc.1972.01750260021004. 4339577

[ref36] Angrist B. Amphetamine psychosis: Clinical variations of the syndrome. In: Amphetamine and its Analogs: Psychopharmacology, Toxicology, and Abuse; Cho A. K., Segel D. S., Eds.; Academic Press: San Diego, 1994; pp 387–414.

[ref37] Bramness J. G.; Gundersen Ø.H.; Guterstam J.; Rognli E. B.; Konstenius M.; Løberg E. M.; Medhus S.; Tanum L.; Franck J. Amphetamine-induced psychosis – a separate diagnostic entity or primary psychosis triggered in the vulnerable?. BMC Psychiatry 2012, 12, 221 10.1186/1471-244X-12-221. 23216941 PMC3554477

[ref38] Geyer M. A.; Callaway C. W. Behavioral pharmacology of ring-substituted amphetamine analogs. In: Amphetamine and its Analogs: Psychopharmacology, Toxicology, and Abuse; Cho A. K., Segel D. S., Eds.; Academic Press: San Diego, 1994; pp 177–208.

[ref39] Wooley D. W. The Biochemical Bases of Psychoses: Or the Serotonin Hypothesis about Mental Diseases; John Wiley and Sons: New York, 1962.

[ref40] Vane J. R. The relative activities of some tryptamine analogues on the isolated rat stomach strip preparation. Br. J. Pharmacol. Chemother. 1959, 14, 87–98. 10.1111/j.1476-5381.1959.tb00933.x. 13651584 PMC1481817

[ref41] Barlow R. B.; Khan I. Actions of some analogues of tryptamine on the isolated rat uterus and on the isolated rat fundus strip preparations. Br. J. Pharmacol. Chemother. 1959, 14, 99–107. 10.1111/j.1476-5381.1959.tb00934.x. 13651585 PMC1481812

[ref42] Johnson C. L.; Green J. P. Molecular orbital studies on tryptamines active on the LSD receptor of the rat fundus strip. Int. J. Quantum Chem. 1974, 8, 159–167. 10.1002/qua.560080720.

[ref43] Glennon R. A.; Gessner P. K. The electronic and serotonin receptor binding affinity properties of *N,N*-dimethyltryptamine analogs. Res. Commun. Chem. Pathol. Pharmacol. 1977, 18, 453–465. 270770

[ref44] Glennon R. A.; Gessner P. K. Serotonin receptor binding affinities of tryptamine analogs. J. Med. Chem. 1979, 22, 428–432. 10.1021/jm00190a014. 430481

[ref45] Glennon R. A. Involvement of serotonin in the action of hallucinogenic agents. In: Neuropharmacology of Serotonin; Green A. R., Ed.; Oxford University Press: Oxford, 1985; pp 253–280.

[ref46] Cheng H. C.; Long J. P.; Nichols D. E.; Barfknecht C. F. Effects of psychotomimetics on vascular strips: studies of methoxylated amphetamines and optical isomers of 2,5-dimethoxy-4-methylamphetamine and 2,5-dimethoxy-4-bromoamphetamine. J. Pharmacol. Exp. Ther. 1974, 188, 114–123. 4809263

[ref47] Dyer D. C.; Nichols D. E.; Rusterholz D. B.; Barfknecht C. F. Comparative effects of stereoisomers of psychotomimetic phenylisopropylamines. Life Sci. 1973, 13, 885–896. 10.1016/0024-3205(73)90079-9. 4766265

[ref48] Nichols D. E.; Shulgin A. T.; Dyer D. C. Directional lipophilic character in a series of psychotomimetic phenethylamine derivatives. Life Sci. 1977, 21, 569–575. 10.1016/0024-3205(77)90099-6. 904435

[ref49] Kier L. B.; Glennon R. A. Psychotomimetic phenalkylamines as serotonin agonists: an SAR analysis. Life Sci. 1978, 22, 1589–1593. 10.1016/0024-3205(78)90053-X. 672414

[ref50] Kier L. B.; Glennon R. A. Progress with several models for the study of hallucinogenic agents. In: QuaSAR: Quantitative Structure Activity Relationships of Analgesics, Narcotic Antagonists, and Hallucinogens; Barnett G., Trsic M., Willette R. E., Eds.; National Institute on Drug Abuse: Washington D.C., 1978; pp 159–185.

[ref51] Seggel M. R.; Yousif M. Y.; Lyon R. A.; Titeler M.; Roth B. L.; Suba E. A.; Glennon R. A. A structure-affinity study of the binding of 4-substituted analogues of 1-(2,5-dimethoxyphenyl)-2-aminopropane at 5-HT_2_ serotonin receptors. J. Med. Chem. 1990, 33, 1032–1036. 10.1021/jm00165a023. 2308135

[ref52] Watts S. W.; Cohen M. L. Characterization of the contractile serotonergic receptor in guinea pig trachea with agonists and antagonists. J. Pharmacol. Exp. Ther. 1992, 260, 1101–1106. 1312158

[ref53] The Peripheral Actions of 5-Hydroxytryptamine; Fozard J. R., Ed.; Oxford Medical Publications: Oxford, 1989.

[ref54] Huang J. T.; Ho B. T. Effect of 2,5-dimethoxy-4-methylamphetamine on heart and smooth muscle contraction. J. Pharm. Pharmacol. 2011, 26, 69–70. 10.1111/j.2042-7158.1974.tb12825.x. 4150941

[ref55] Standridge R. T.; Howell H. G.; Gylys J. A.; Partyka R. A.; Shulgin A. T. Phenylakylamines with potential psychotherapeutic utility. 1. 2-Amino-1-(2,5-dimethoxy-4-methylphenyl)butane. J. Med. Chem. 1976, 19, 1400–1404. 10.1021/jm00234a010. 1003425

[ref56] Glennon R. A.; Liebowitz S. M.; Mack E. C. Serotonin receptor binding affinities of several hallucinogenic phenylalkylamine and *N,N*-dimethyltryptamine analogues. J. Med. Chem. 1978, 21, 822–825. 10.1021/jm00206a022. 278843

[ref57] Glennon R. A.; Liebowitz S. M.; Anderson G. M. 3rd Serotonin receptor affinities of psychoactive phenalkylamine analogues. J. Med. Chem. 1980, 23, 294–299. 10.1021/jm00177a017. 7365744

[ref58] Glennon R. A.; Liebowitz S. M.; Leming-Doot D.; Rosecrans J. A. Desmethyl analogues of psychoactive methoxyphenalkylamines: synthesis and serotonin receptor affinities. J. Med. Chem. 1980, 23, 990–994. 10.1021/jm00183a006. 7411554

[ref59] Glennon R. A.; Doot D. L.; Young R. DOM and related 2,5-dimethoxy-4-alkylphenylisopropylamines: behavioral and serotonin receptor properties. Pharmacol., Biochem. Behav. 1981, 14, 287–292. 10.1016/0091-3057(81)90392-0. 7232455

[ref60] Domelsmith L. N.; Eaton T. A.; Houk K. N.; Anderson G. M. 3rd; Glennon R. A.; Shulgin A. T.; Castagnoli N. Jr.; Kollman P. A. Photoelectron spectra of psychotropic drugs. 6. Relationships between the physical properties and pharmacological actions of amphetamine analogues. J. Med. Chem. 1981, 24, 1414–1421. 10.1021/jm00144a009. 7310818

[ref61] Glennon R. A.; Young R.; Benington F.; Morin R. D. Behavioral and serotonin receptor properties of 4-substituted derivatives of the hallucinogen 1-(2,5-dimethoxyphenyl)-2-aminopropane. J. Med. Chem. 1982, 25, 1163–1168. 10.1021/jm00352a013. 7143352

[ref62] Drug Discrimination: Applications to Medicinal Chemistry and Drug Studies; Glennon R. A., Young R., Eds; John Wiley and Sons: Hoboken, NJ, 2011.

[ref63] Glennon R. A.; Rosecrans J. A.; Young R. Drug-induced discrimination: a description of the paradigm and a review of its specific application to the study of hallucinogenic agents. Med. Res. Rev. 1983, 3, 289–340. 10.1002/med.2610030305. 6350763

[ref64] Winter J. C. The Effects of 2,5-dimethoxy-4-methylamphetamine (DOM), 2,5-dimethoxy-4-ethylamphetamine (DOET), d-amphetamine, and cocaine in rats trained with mescaline as a discriminative stimulus. Psychopharmacologia (Berl.) 1975, 44, 29–32. 10.1007/BF00421179. 1197576

[ref65] Winter J. C. Stimulus properties of phenethylamine hallucinogens and lysergic acid diethylamide: the role of 5-hydroxytryptamine. J. Pharmacol. Exp. Ther. 1978, 204, 416–423. 621672

[ref66] Winter J. C. Hallucinogens as discriminative stimuli in animals: LSD, phenethylamines, and tryptamines. Psychopharmacology 2009, 203, 251–263. 10.1007/s00213-008-1356-8. 18979087

[ref67] Appel J. B.; White F. J.; Holohean A. M. Analyzing mechanism(s) of hallucinogenic drug action with drug discrimination procedures. Neurosci. Biobehav. Rev. 1982, 6, 529–536. 10.1016/0149-7634(82)90036-7. 7177512

[ref68] Glennon R. A.; Rosecrans J. A.; Young R.; Gaines J. Hallucinogens as a discriminative stimuli: generalization of DOM to a 5-methoxy-*N,N*-dimethyltryptamine stimulus. Life Sci. 1979, 24, 993–997. 10.1016/0024-3205(79)90317-5. 286864

[ref69] Glennon R. A.; Rosecrans J. A.; Young R. The use of the drug discrimination paradigm for studying hallucinogenic agents. In: Drug Discrimination: Applications In CNS Pharmacology; Colpaert F. C., Slangen J. L., Eds.; Elsevier Biomedical: Amsterdam, 1982; pp 69–95.

[ref70] Glennon R. A. Classical hallucinogens. In: Handbook of Experimental Pharmacology: Pharmacological Aspects of Drug Dependence; Schuster C. R., Kuhar M. J., Eds.; Springer Verlag: Basel, Switzerland, 1996; pp 343–371.

[ref71] Glennon R. A.; Rosecrans J. A. Speculations on the mechanism of action of hallucinogenic indolealkylamines. Neurosci. Biobehav. Rev. 1981, 5, 197–207. 10.1016/0149-7634(81)90002-6. 7022271

[ref72] Young R.; Rosecrans J. A.; Glennon R. A. Comparative discriminative stimulus effects of 5-methoxy-*N*,*N*-dimethyltryptamine and LSD. Life Sci. 1982, 30, 2057–2062. 10.1016/0024-3205(82)90446-5. 7109835

[ref73] Young R.; Rosecrans J. A.; Glennon R. A. Behavioral effects of 5-methoxy-*N*,*N*-dimethyltryptamine and dose-dependent antagonism by BC-105. Psychopharmacology (Berl.) 1983, 80, 156–160. 10.1007/BF00427960. 6410445

[ref74] Young R.; Rosecrans J. A.; Glennon R. A. Further studies on the dose-dependent stimulus properties of 5-methoxy-*N,N*-dimethyltryptamine. Pharmacol., Biochem. Behav. 1986, 25, 1207–1210. 10.1016/0091-3057(86)90113-9. 3809222

[ref75] Stolerman I. P.; Childs E.; Ford M. M.; Grant K. A. Role of training dose in drug discrimination: a review. Behav. Pharmacol. 2011, 22, 415–429. 10.1097/FBP.0b013e328349ab37. 21808191 PMC3155633

[ref76] White F. J.; Appel J. B. Training dose as a factor in LSD-saline discrimination. Psychopharmacology (Berl.) 1982, 76, 20–25. 10.1007/BF00430748. 6805003

[ref77] Silverman P. B.; Ho B. T. The discriminative stimulus properties of 2,5-dimethoxy-4-methylamphetamine (DOM): differentiation from amphetamine. Psychopharmacology (Berl.) 1980, 68, 209–215. 10.1007/BF00428105. 6771804

[ref78] Young R.; Glennon R. A.; Rosecrans J. A. Discriminative stimulus properties of the hallucinogenic agent DOM. Commun. Psychopharmacol. 1980, 4, 501–506. 7297054

[ref79] Shulgin A.; Shulgin A. TIHKAL; Joy D., Ed.; Transform Press: Berkeley, CA, 1997.

[ref80] Gessner P. K.; Godse D. D.; Krull A. H.; McMullan J. M. Structure-activity relationships among 5-methoxy-N:N-dimethyltryptamine, 4-hydroxy-N:N-dimethyltryptamine (psilocin) and other substituted tryptamines. Life Sci. 1968, 7, 267–277. 10.1016/0024-3205(68)90200-2. 5641719

[ref81] Glennon R. A.; Young R.; Rosecrans J. A.; Kallman M. J. Hallucinogenic agents as discriminative stimuli: a correlation with serotonin receptor affinities. Psychopharmacology (Berl.) 1980, 68, 155–158. 10.1007/BF00432133. 6776558

[ref82] Glennon R. A.; Young R.; Jacyno J. M.; Slusher M.; Rosecrans J. A. DOM-stimulus generalization to LSD and other hallucinogenic indolealkylamines. Eur. J. Pharmacol. 1983, 86, 453–459. 10.1016/0014-2999(83)90196-6. 6572591

[ref83] Glennon R. A.; Young R.; Rosecrans J. A. Discriminative stimulus properties of DOM and several molecular modifications. Pharmacol., Biochem. Behav. 1982, 16, 553–556. 10.1016/0091-3057(82)90413-0. 7071088

[ref84] Glennon R. A.; Young R.; Jacyno J. M. Indolealkylamine and phenalkylamine hallucinogens. Effect of alpha-methyl and N-methyl substituents on behavioral activity. Biochem. Pharmacol. 1983, 32, 1267–1273. 10.1016/0006-2952(83)90281-2. 6573879

[ref85] Huang J. T.; Ho B. T. Discriminative stimulus properties of d-amphetamine and related compounds in rats. Pharmacol., Biochem. Behav. 1974, 2, 669–673. 10.1016/0091-3057(74)90036-7. 4431829

[ref86] Young R.; Glennon R. A. Discriminative stimulus properties of amphetamine and structurally related phenalkylamines. Med. Res. Rev. 1986, 6, 99–130. 10.1002/med.2610060105. 3512936

[ref87] Glennon R. A. Stimulus properties of hallucinogenic phenalkylamines and related designer drugs: formulation of structure-activity relationships. In: Pharmacology and Toxicology of Amphetamine and Related Designer Drugs; Ashgar R., DeSouza E., Eds.; NIDA Research Monograph 94; National Institute on Drug Abuse, U.S. Government Printing Office: Washington, DC, 1989; pp 43–67. PMID: 2575229. 2575229

[ref88] Glennon R. A. Discriminative stimulus properties of hallucinogens and related designer drugs. In: Drug Discrimination: Applications to Drug Abuse Research; Glennon R. A., Jarbe T. U. C., Frankenheim J., Eds.; National Institute of Drug Abuse (Publication number ADM92-1978): Rockville, MD, 1991; pp 25–44. 1369672

[ref89] Glennon R. A.; Young R. Drug discrimination and in vivo structure-activity relationships. In: Drug Discrimination: Applications to Medicinal Chemistry and Drug Studies; Glennon R. A., Young R., Eds.; John Wiley and Sons: Hoboken, NJ, 2011; pp 163–181.

[ref90] Nichols D. E.; Glennon R. A. Medicinal chemistry and structure-activity relationships of hallucinogens. In: Hallucinogens: Neurochemical, Behavioral, and Clinical Properties; Jacobs B. L., Ed.; Raven Press: New York, 1984; pp 95–142.

[ref91] Nichols D. E. Chemistry and structure–activity relationships of psychedelics. In: Behavioral Neurobiology of Psychedelic Drugs. Current Topics in Behavioral Neurosciences, Vol 36.; Halberstadt A. L., Vollenweider F. X., Nichols D. E., Eds.; Springer: Berlin, Heidelberg, 2017. 10.1007/7854_2017_475. 28401524

[ref92] Nichols D. E. Medicinal chemistry and structure-activity relationships. In: Amphetamine and its Analogs: Psychopharmacology, Toxicology, and Abuse; Cho A. K., Segel D. S., Eds.; Academic Press: San Diego, 1994; pp 3–41.

[ref93] Corne S. J.; Pickering R. W. A possible correlation between drug-induced hallucinations in man and a behavioural response in mice. Psychopharmacologia 1967, 11, 65–78. 10.1007/BF00401509. 5302272

[ref94] Glennon R. A.; Lucki I. Behavioral models of serotonin receptor activation. In: The Serotonin Receptors; Sanders-Bush E., Ed.; Humana Press: Clifton, NJ, 1988; pp 253–293.

[ref95] Glennon R. A.; Darmani N. A.; Martin B. R. Multiple populations of serotonin receptors may modulate the behavioral effects of serotonergic agents. Life Sci. 1991, 48, 2493–2498. 10.1016/0024-3205(91)90603-9. 2046474

[ref96] Halberstadt A. L.; Geyer M. A. Effect of hallucinogens on unconditioned behavior. Curr. Top. Behav. Neurosci. 2016, 36, 159–199. 10.1007/7854_2016_466. PMC5787039 28224459

[ref97] Halberstadt A. L.; Nichols D. E. Serotonin and serotonin receptors in hallucinogen action. Chapter 43. Handbook of Behavioral Neuroscience, Vol. 31;Müller C. P., Cunningham K. A., Eds.; Elsevier, 2020; pp 843–863. 10.1016/B978-0-444-64125-0.00043-8.

[ref98] Darmani N. A.; Martin B. R.; Pandey U.; Glennon R. A. Do functional relationships exist between 5-HT_1A_ and 5-HT_2_ receptors?. Pharmacol., Biochem. Behav. 1990, 36, 901–906. 10.1016/0091-3057(90)90098-3. 2145593

[ref99] Darmani N. A.; Martin B. R.; Glennon R. A. Withdrawal from chronic treatment with (±)DOI causes super-sensitivity to 5-HT_2_ receptor-induced head-twitch behaviour in mice. Eur. J. Pharmacol. 1990, 186, 115–118. 10.1016/0014-2999(90)94066-7. 2282932

[ref100] Canal C. E.; Morgan D. Head-twitch response in rodents induced by the hallucinogen 2,5-dimethoxy-4-iodoamphetamine: a comprehensive history, a re-evaluation of mechanisms, and its utility as a model. Drug Test. Anal. 2012, 4, 556–576. 10.1002/dta.1333. 22517680 PMC3722587

[ref101] Oppong-Damoah A.; Curry K. E.; Blough B. E.; Rice K. C.; Murnane K. S. Effects of the synthetic psychedelic 2,5-dimethoxy-4-iodoamphetamine (DOI) on ethanol consumption and place conditioning in male mice. Psychopharmacology (Berl.) 2019, 236, 3567–3578. 10.1007/s00213-019-05328-7. 31309240 PMC6895420

[ref102] Darmani N. A.; Martin B. R.; Glennon R. A. Behavioral evidence for differential adaptation of the serotonergic system after acute and chronic treatment with (±)-1-(2,5-dimethoxy-4-iodophenyl)-2-aminopropane (DOI) or ketanserin. J. Pharmacol. Exp. Ther. 1992, 262, 692–698. 1501117

[ref103] Darmani N. A. Further evidence for enigmas in adaptation mechanisms for the DOI-induced behaviors. Pharmacol., Biochem. Behav. 1992, 43, 765–770. 10.1016/0091-3057(92)90406-6. 1448470

[ref104] Schreiber R.; Brocco M.; Audinot V.; Gobert A.; Veiga S.; Millan M. J. (1-(2,5-Dimethoxy-4-iodophenyl)-2-aminopropane)-induced head-twitches in the rat are mediated by 5-hydroxytryptamine (5-HT) 2A receptors: modulation by novel 5-HT_2A/2C_ antagonists, D1 antagonists and 5-HT_1A_ agonists. J. Pharmacol. Exp. Ther. 1995, 273, 101–112. 7714755

[ref105] Berendsen H. H.; Broekkamp C. L. Behavioural evidence for functional interactions between 5-HT-receptor subtypes in rats and mice. Br. J. Pharmacol. 1990, 101, 667–673. 10.1111/j.1476-5381.1990.tb14138.x. 2150180 PMC1917735

[ref106] Darmani N. A.; Mock O. B.; Towns L. C.; Gerdes C. F. The head-twitch response in the least shrew (Cryptotis parva) is a 5-HT_2_- and not a 5-HT_1C_-mediated phenomenon. Pharmacol., Biochem. Behav. 1994, 48, 383–396. 10.1016/0091-3057(94)90542-8. 8090805

[ref107] Kulkarni A. S. Scratching response induced in mice by mescaline and related amphetamine derivatives. Biol. Psychiatry 1973, 6, 177–180. 4709133

[ref108] Yim G. K. W.; Prah T. E.; Pfister W. R.; Nichols D. E. An economical screen for phenethylamine-type hallucinogens: mouse ear scratching. Commun. Psychopharmacol. 1979, 3, 173–178. 574070

[ref109] Darmani N. A.; Martin B. R.; Pandey U.; Glennon R. A. Pharmacological characterization of ear-scratch response in mice as a behavioral model for selective 5-HT_2_ receptor agonists and evidence for 5-HT_1B_- and 5-HT_2_-receptor interactions. Pharmacol., Biochem. Behav. 1990, 37, 95–99. 10.1016/0091-3057(90)90047-L. 2263671

[ref110] Canal C. E.; Olaghere da Silva U. B.; Gresch P. J.; Watt E. E.; Sanders-Bush E.; Airey D. C. The serotonin 2C receptor potently modulates the head-twitch response in mice induced by a phenethylamine hallucinogen. Psychopharmacology (Berl.) 2010, 209, 163–174. 10.1007/s00213-010-1784-0. 20165943 PMC2868321

[ref111] Fantegrossi W. E.; Simoneau J.; Cohen M. S.; Zimmerman S. M.; Henson C. M.; Rice K. C.; Woods J. H. Interaction of 5-HT_2A_ and 5-HT_2C_ receptors in *R*(−)-2,5-dimethoxy-4-iodoamphetamine-elicited head twitch behavior in mice. J. Pharmacol. Exp. Ther. 2010, 335, 728–734. 10.1124/jpet.110.172247. 20858706 PMC2993545

[ref112] Canal C. E.; Booth R. G.; Morgan D. Support for 5-HT_2C_ receptor functional selectivity in vivo utilizing structurally diverse, selective 5-HT_2C_ receptor ligands and the 2,5-dimethoxy-4-iodoamphetamine elicited head-twitch response model. Neuropharmacology 2013, 70, 112–121. 10.1016/j.neuropharm.2013.01.007. 23353901 PMC3754837

[ref113] Custodio R. J. P.; Ortiz D. M.; Lee H. J.; Sayson L. V.; Kim M.; Lee Y. S.; Kim K. M.; Cheong J. H.; Kim H. J. Serotonin 2C receptors are also important in head-twitch responses in male mice. Psychopharmacology (Berl.) 2023, Oct 26. 10.1007/s00213-023-06482-9. Epub ahead of print. PMID: 37882810. 37882810

[ref114] Wallach J.; Cao A. B.; Calkins M. M.; Heim A. J.; Lanham J. K.; Bonniwell E. M.; Hennessey J. J.; Bock H. A.; Anderson E. I.; Sherwood A. M.; Morris H.; de Klein R.; Klein A. K.; Cuccurazzu B.; Gamrat J.; Fannana T.; Zauhar R.; Halberstadt A. L.; McCorvy J. D. Identification of 5-HT_2A_ receptor signaling pathways responsible for psychedelic potential. bioRxiv [Preprint]. **2023** Jul 31:2023.07.29.551106. 10.1101/2023.07.29.551106. PMID: 37577474; PMCID: PMC10418054. PMC10724237 38102107

[ref115] Willins D. L.; Meltzer H. Y. Direct injection of 5-HT_2A_ receptor agonists into the medial prefrontal cortex produces a head-twitch response in rats. J. Pharmacol. Exp. Ther. 1997, 282, 699–706. 9262333

[ref116] Sakaue M.; Ago Y.; Sowa C.; Sakamoto Y.; Nishihara B.; Koyama Y.; Baba A.; Matsuda T. Modulation by 5-HT_2A_ receptors of aggressive behavior in isolated mice. Jpn. J. Pharmacol. 2002, 89, 89–92. 10.1254/jjp.89.89. 12083749

[ref117] Egashira N.; Mishima K.; Uchida T.; Hasebe N.; Nagai H.; Mizuki A.; Iwasaki K.; Ishii H.; Nishimura R.; Shoyama Y.; Fujiwara M. Anandamide inhibits the DOI-induced head-twitch response in mice. Psychopharmacology (Berl.) 2004, 171, 382–389. 10.1007/s00213-003-1611-y. 14586538

[ref118] Hayslett R. L.; Tizabi Y. Effects of donepezil on DOI-induced head twitch response in mice: implications for Tourette syndrome. Pharmacol., Biochem. Behav. 2003, 76, 409–415. 10.1016/j.pbb.2003.08.015. 14643839

[ref119] Miyata S.; Hirano S.; Kamei J. Diabetes inhibits the DOI-induced head-twitch response in mice. Psychopharmacology (Berl.) 2004, 177, 224–229. 10.1007/s00213-004-1942-3. 15290007

[ref120] Nakagawasai O.; Murata A.; Arai Y.; Ohba A.; Wakui K.; Mitazaki S.; Niijima F.; Tan-No K.; Tadano T. Enhanced head-twitch response to 5-HT-related agonists in thiamine-deficient mice. J. Neural Transm. (Vienna) 2007, 114, 1003–1010. 10.1007/s00702-007-0655-2. 17372673

[ref121] Hazama K.; Hayata-Takano A.; Uetsuki K.; Kasai A.; Encho N.; Shintani N.; Nagayasu K.; Hashimoto R.; Reglodi D.; Miyakawa T.; Nakazawa T.; Baba A.; Hashimoto H. Increased behavioral and neuronal responses to a hallucinogenic drug in PACAP heterozygous mutant mice. PLoS One 2014, 9, e89153 10.1371/journal.pone.0089153. 24586556 PMC3930680

[ref122] Malik M.; Rangel-Barajas C.; Mach R. H.; Luedtke R. R. The effect of the sigma-1 receptor selective compound LS-1–137 on the DOI-induced head twitch response in mice. Pharmacol., Biochem. Behav. 2016, 148, 136–144. 10.1016/j.pbb.2016.07.001. 27397487

[ref123] Jaster A. M.; González-Maeso J. Automated detection of psychedelic-induced head-twitch response in mice. Methods Mol. Biol. 2023, 2687, 65–76. 10.1007/978-1-0716-3307-6_6. 37464163

[ref124] Halberstadt A. L.; Geyer M. A. Characterization of the head-twitch response induced by hallucinogens in mice: detection of the behavior based on the dynamics of head movement. Psychopharmacology (Berl.) 2013, 227, 727–739. 10.1007/s00213-013-3006-z. 23407781 PMC3866102

[ref125] de la Fuente Revenga M.; Shin J. M.; Vohra H. Z.; Hideshima K. S.; Schneck M.; Poklis J. L.; González-Maeso J. Fully automated head-twitch detection system for the study of 5-HT_2A_ receptor pharmacology in vivo. Sci. Rep. 2019, 9, 14247 10.1038/s41598-019-49913-4. 31582824 PMC6776537

[ref126] Halberstadt A. L.; Chatha M.; Klein A. K.; Wallach J.; Brandt S. D. Correlation between the potency of hallucinogens in the mouse head-twitch response assay and their behavioral and subjective effects in other species. Neuropharmacology 2020, 167, 107933 10.1016/j.neuropharm.2019.107933. 31917152 PMC9191653

[ref127] Gewirtz J. C.; Marek G. J. Behavioral evidence for interactions between a hallucinogenic drug and group II metabotropic glutamate receptors. Neuropsychopharmacology 2000, 23, 569–576. 10.1016/S0893-133X(00)00136-6. 11027922

[ref128] Kłodzinska A.; Bijak M.; Tokarski K.; Pilc A. Group II mGlu receptor agonists inhibit behavioural and electrophysiological effects of DOI in mice. Pharmacol., Biochem. Behav. 2002, 73, 327–332. 10.1016/S0091-3057(02)00845-6. 12117586

[ref129] Zhai Y.; George C. A.; Zhai J.; Nisenbaum E. S.; Johnson M. P.; Nisenbaum L. K. Group II metabotropic glutamate receptor modulation of DOI-induced c-fos mRNA and excitatory responses in the cerebral cortex. Neuropsychopharmacology 2003, 28, 45–52. 10.1038/sj.npp.1300013. 12496939

[ref130] Zhang C.; Marek G. J. AMPA receptor involvement in 5-hydroxytryptamine2A receptor-mediated pre-frontal cortical excitatory synaptic currents and DOI-induced head shakes. Prog. Neuropsychopharmacol. Biol. Psychiatry 2008, 32, 62–71. 10.1016/j.pnpbp.2007.07.009. 17728034

[ref131] Peroutka S. J.; Snyder S. H. Multiple serotonin receptors: differential binding of [^3^H]5-hydroxytryptamine, [^3^H]lysergic acid diethylamide and [^3^H]spiroperidol. Mol. Pharmacol. 1979, 16, 687–699. 530254

[ref132] Barnes N. M.; Ahern G. P.; Becamel C.; Bockaert J.; Camilleri M.; Chaumont-Dubel S.; Claeysen S.; Cunningham K. A.; Fone K. C.; Gershon M.; Di Giovanni G.; Goodfellow N. M.; Halberstadt A. L.; Hartley R. M.; Hassaine G.; Herrick-Davis K.; Hovius R.; Lacivita E.; Lambe E. K.; Leopoldo M.; Levy F. O.; Lummis S. C. R.; Marin P.; Maroteaux L.; McCreary A. C.; Nelson D. L.; Neumaier J. F.; Newman-Tancredi A.; Nury H.; Roberts A.; Roth B. L.; Roumier A.; Sanger G. J.; Teitler M.; Sharp T.; Villalón C. M.; Vogel H.; Watts S. W.; Hoyer D. International Union of Basic and Clinical Pharmacology. CX. Classification of receptors for 5-hydroxytryptamine; pharmacology and function. Pharmacol. Rev. 2021, 73, 310–520. 10.1124/pr.118.015552. 33370241 PMC7770494

[ref133] Glennon R. A.; Raghupathi R.; Bartyzel P.; Teitler M.; Leonhardt S. Binding of phenylalkylamine derivatives at 5-HT_1C_ and 5-HT_2_ serotonin receptors: evidence for a lack of selectivity. J. Med. Chem. 1992, 35, 734–740. 10.1021/jm00082a014. 1542100

[ref134] Nelson D. L.; Lucaites V. L.; Wainscott D. B.; Glennon R. A. Comparisons of hallucinogenic phenylisopropylamine binding affinities at cloned human 5-HT_2A_, 5-HT_2B_ and 5-HT_2C_ receptors. Naunyn-Schmiedebergs Arch. Pharmacol. 1999, 359, 1–6. 10.1007/PL00005315. 9933142

[ref135] Hemanth P.; Nistala P.; Nguyen V. T.; Eltit J. M.; Glennon R. A.; Dukat M. Binding and functional structure-activity similarities of 4-substituted 2,5-dimethoxyphenyl isopropylamine analogues at 5-HT_2A_ and 5-HT_2B_ serotonin receptors. Front. Pharmacol. 2023, 14, 1101290 10.3389/fphar.2023.1101290. 36762110 PMC9902381

[ref136] Jaster A. M.; González-Maeso J. Mechanisms and molecular targets surrounding the potential therapeutic effects of psychedelics. Mol. Psychiatry 2023, 28, 3595–3612. 10.1038/s41380-023-02274-x. 37759040 PMC11078317

[ref137] Glennon R. A.; Westkaemper R. B.; Bartyzel P. Medicinal chemistry of serotonergic agents. In: Serotonin Receptor Subtypes: Basic and Clinical Aspects; Peroutka S. J., Ed.; Wiley-Liss: New York, 1991; pp 19–64.

[ref138] Glennon R. A.; Dukat M. Serotonin receptors and their ligands: a lack of selective agents. Pharmacol., Biochem. Behav. 1991, 40, 1009–1017. 10.1016/0091-3057(91)90121-H. 1816555

[ref139] Glennon R. A.; Peroutka S. J.; Dukat M. Binding characteristics of a quaternary amine analog of serotonin: 5-HTQ. In Serotonin: Molecular Biology, Receptors and Functional Effects; Fozard J. R., Saxena P. R., Eds.; Birkhauser-Verlag: Basel, Switzerland, 1991; pp 186–192.

[ref140] Glennon R. A. Functional/clinical significance of 5-hydroxytryptamine binding sites. In: Serotonin: From Cell Biology to Pharmacology and Therapeutics; Paoletti R., Vanhoutte P. M., Brunello N., Maggi F. M., Eds.; Kluwer Academic Publishers: Dordrecht, 1990; pp 259–264.

[ref141] Shannon M.; Battaglia G.; Glennon R. A.; Titeler M. 5-HT_1_ and 5-HT_2_ binding properties of derivatives of the hallucinogen 1-(2,5-dimethoxyphenyl)-2-aminopropane (2,5-DMA). Eur. J. Pharmacol. 1984, 102, 23–29. 10.1016/0014-2999(84)90333-9. 6479216

[ref142] Glennon R. A.; McKenney J. D.; Lyon R. A.; Titeler M. 5-HT_1_ and 5-HT_2_ binding characteristics of 1-(2,5-dimethoxy-4-bromophenyl)-2-aminopropane Analogues. J. Med. Chem. 1986, 29, 194–199. 10.1021/jm00152a005. 3950904

[ref143] Glennon R. A.; Titeler M.; Young R. A. Structure-Activity Relationships and Mechanism of Action of Hallucinogenic Agents Based on Drug Discrimination and Radioligand Binding Studies. Psychopharmacol. Bull. 1986, 22, 953–958. 3025916

[ref144] Glennon R. A.; Seggel M. R. Interaction of phenylisopropylamines with 5-HT_2_ receptors: A QSAR analysis. In: Bioactive Mechanisms; Magee P. S., Henry D. R., Block J. H.; American Chemical Society: Washington, DC, 1989; pp 264–280.

[ref145] Glennon R. A.; Dukat M.; Grella B.; Hong S.; Costantino L.; Teitler M.; Smith C.; Egan C.; Davis K.; Mattson M. V. Binding of beta-carbolines and related agents at serotonin (5-HT_2_ and 5-HT_1A_), dopamine (D2) and benzodiazepine receptors. Drug Alcohol Depend. 2000, 60, 121–132. 10.1016/S0376-8716(99)00148-9. 10940539

[ref146] Dowd C. S.; Herrick-Davis K.; Egan C.; DuPre A.; Smith C.; Teitler M.; Glennon R. A. 1-[4-(3-Phenylalkyl)phenyl]-2-aminopropanes as 5-HT_2A_ partial agonists. J. Med. Chem. 2000, 43, 3074–3084. 10.1021/jm9906062. 10956215

[ref147] Marcher-Rørsted E.; Halberstadt A. L.; Klein A. K.; Chatha M.; Jademyr S.; Jensen A. A.; Kristensen J. L. Investigation of the 2,5-dimethoxy motif in phenethylamine serotonin 2A receptor agonists. ACS Chem. Neurosc. 2020, 11, 1238–1244. 10.1021/acschemneuro.0c00129. 32212672

[ref148] Rangisetty J. B.; Dukat M.; Dowd C. S.; Herrick-Davis K.; DuPre A.; Gadepalli S.; Teitler M.; Kelley C. R.; Sharif N. A.; Glennon R. A. 1-[2-Methoxy-5-(3-phenylpropyl)]-2-aminopropane unexpectedly shows 5-HT_2A_ serotonin receptor affinity and antagonist character. J. Med. Chem. 2001, 44, 3283–3291. 10.1021/jm0100739. 11563927

[ref149] Marona-Lewicka D.; Rhee G. S.; Sprague J. E.; Nichols D. E. Psychostimulant-like effects of p-fluoroamphetamine in the rat. Eur. J. Pharmacol. 1995, 287, 105–113. 10.1016/0014-2999(95)00478-5. 8749023

[ref150] Peddi S.; Roth B. L.; Glennon R. A.; Westkaemper R. B. Ring substituted analogues of 5-aminomethyl-10,11-dihydro-dibenzo[a,d]cycloheptene (AMDH): potential modes of binding to the 5-HT_2A_ receptor. Bioorg. Med. Chem. Lett. 2003, 13, 2565–2568. 10.1016/S0960-894X(03)00504-3. 12852967

[ref151] Runyon S. P.; Mosier P. D.; Roth B. L.; Glennon R. A.; Westkaemper R. B. Potential modes of interaction of 9-aminomethyl-9,10-dihydroanthracene (AMDA) derivatives with the 5-HT_2A_ receptor: a ligand structure-affinity relationship, receptor mutagenesis and receptor modeling investigation. J. Med. Chem. 2008, 51, 6808–6828. 10.1021/jm800771x. 18847250 PMC3088499

[ref152] Titeler M.; Herrick K.; Lyon R. A.; McKenney J. D.; Glennon R. A. [^3^H]DOB: a specific radioligand for 5-HT_2_ serotonin receptors. Eur. J. Pharmacol. 1985, 117, 145–146. 10.1016/0014-2999(85)90486-8. 4085543

[ref153] Titeler M.; Lyon R. A.; Davis K. H.; Glennon R. A. Selectivity of serotonergic drugs for multiple brain serotonin receptors: role of [^3^H]-4-bromo-2,5-dimethoxyphenylisopropylamine ([^3^H]DOB), a 5-HT_2_ agonist radioligand. Biochem. Pharmacol. 1987, 36, 3265–3271. 10.1016/0006-2952(87)90643-5. 3663239

[ref154] Pierce P. A.; Peroutka S. J. Hallucinogenic drug interactions with neurotransmitter receptor binding sites in human cortex. Psychopharmacology (Berl.) 1989, 97, 118–122. 10.1007/BF00443425. 2540505

[ref155] Lyon R. A.; Titeler M. Pharmacology and biochemistry of the 5-HT_2_ receptor. In: The Serotonin Receptors; Sanders-Bush E., Ed.; Humana Press: Clifton, NJ, 1988; pp 59–88.

[ref156] Glennon R. A.; Seggel M. R.; Soine W. H.; Herrick-Davis K.; Lyon R. A.; Titeler M. [^125^I]-1-(2,5-Dimethoxy-4-iodophenyl)-2-aminopropane: an iodinated radioligand that specifically labels the agonist high-affinity state of 5-HT_2_ serotonin receptors. J. Med. Chem. 1988, 31, 5–7. 10.1021/jm00396a003. 3336031

[ref157] Johnson M. P.; Hoffman A. J.; Nichols D. E.; Mathis C. A. Binding to the serotonin 5-HT_2_ receptor by the enantiomers of ^125^I-DOI. Neuropharmacology 1987, 26, 1803–1806. 10.1016/0028-3908(87)90138-9. 3437942

[ref158] McKenna D. J.; Peroutka S. J. Differentiation of 5-hydroxytryptamine_2_ receptor subtypes using ^125^I-R-(−)2,5-dimethoxy-4-iodo-phenylisopropylamine and ^3^H-ketanserin. J. Neurosci. 1989, 9, 3482–3490. 10.1523/JNEUROSCI.09-10-03482.1989. 2795135 PMC6569888

[ref159] McKenna D. J.; Saavedra J. M. Autoradiography of LSD and 2,5-dimethoxyphenylisopropylamine psychotomimetics demonstrates regional, specific cross-displacement in the rat brain. Eur. J. Pharmacol. 1987, 142, 313–315. 10.1016/0014-2999(87)90121-X. 3691644

[ref160] Branchek T.; Adham N.; Macchi M.; Kao H. T.; Hartig P. R. [^3^H]-DOB (4-bromo-2,5-dimethoxyphenylisopropylamine) and [^3^H] ketanserin label two affinity states of the cloned human 5-hydroxytryptamine2 receptor. Mol. Pharmacol. 1990, 38, 604–609. 2233697

[ref161] Leonhardt S.; Titeler M. Serotonin 5-HT_2_ receptors: two states versus two subtypes. J. Neurochem. 1989, 53, 316–318. 10.1111/j.1471-4159.1989.tb07334.x. 2723661

[ref162] Teitler M.; Leonhardt S.; Weisberg E. L.; Hoffman B. J. 4-[^125^I]Iodo-(2,5-dimethoxy)phenylisopropylamine and [^3^H]ketanserin labeling of 5-hydroxytryptamine2 (5HT_2_) receptors in mammalian cells transfected with a rat 5HT_2_ cDNA: evidence for multiple states and not multiple 5HT_2_ receptor subtypes. Mol. Pharmacol. 1990, 38, 594–598. 2233696

[ref163] Fitzgerald L. W.; Conklin D. S.; Krause C. M.; Marshall A. P.; Patterson J. P.; Tran D. P.; Iyer G.; Kostich W. A.; Largent B. L.; Hartig P. R. High-affinity agonist binding correlates with efficacy (intrinsic activity) at the human serotonin 5-HT_2A_ and 5-HT_2C_ receptors: evidence favoring the ternary complex and two-state models of agonist action. J. Neurochem. 1999, 72, 2127–2134. 10.1046/j.1471-4159.1999.0722127.x. 10217294

[ref164] Appel N. M.; Mitchell W. M.; Garlick R. K.; Glennon R. A.; Teitler M.; De Souza E. B. Autoradiographic characterization of (±)-1-(2,5-dimethoxy-4-[^125^I] iodophenyl)-2-aminopropane ([^125^I]DOI) binding to 5-HT_2_ and 5-HT_1C_ receptors in rat brain. J. Pharmacol. Exp. Ther. 1990, 255, 843–857. 2243353

[ref165] Saavedra J. M.; Himeno A. Autoradiographic studies of 5HT_2_ receptors. Adv. Exp. Med. Biol. 1991, 294, 107–113. 10.1007/978-1-4684-5952-4_9. 1772060

[ref166] Appel N. M. Autoradiographic approaches to studying hallucinogens or other drugs. In: Hallucinogens: An Update; Lin G. C., Glennon R. A., Eds.; NIDA Research Monograph 146; National Institute on Drug Abuse: Rockville, 1994; pp 214–240. 8742801

[ref167] Nazarali A. J.; McKenna D. J.; Saavedra J. M. Autoradiographic localization of 5HT_2_ receptors in rat brain using [^125^I]-DOI, a selective psychotomimetic radioligand. Prog. Neuropsychopharmacol. Biol. Psychiatry 1989, 13, 573–581. 10.1016/0278-5846(89)90149-8. 2748882

[ref168] McKenna D. J.; Nazarali A. J.; Hoffman A. J.; Nichols D. E.; Mathis C. A.; Saavedra J. M. Common receptors for hallucinogens in rat brain: a comparative autoradiographic study using [^125^I]LSD and [^125^I]DOI, a new psychotomimetic radioligand. Brain Res. 1989, 476, 45–56. 10.1016/0006-8993(89)91535-7. 2536576

[ref169] Sargent T. 3rd; Budinger T. F.; Braun G.; Shulgin A. T.; Braun U. An iodinated catecholamine congener for brain imaging and metabolic studies. J. Nucl. Med. 1978, 19, 71–76. 413888

[ref170] Lee K. S.; Schiek C.; Glennon R. A.; Jones D. W.; Gorey J. J.; Knable M. B.; Coppola R.; Rice K. C.; Weinberger D. R. Synthesis of novel radioiodinated 5-HT_2_ analogs for SPECT imaging of serotonin receptors. J. Nucl. Med. 1997, 38, 271P.

[ref171] Zea-Ponce Y.; Kegeles L. S.; Guo N.; Raskin L.; Bakthavachalam V.; Laruelle M. Pharmacokinetics and brain distribution in non human primate of *R*(−)[^123^I]DOI, a 5HT(2A/2C) serotonin agonist. Nucl. Med. Biol. 2002, 29, 575–583. 10.1016/S0969-8051(02)00306-2. 12088728

[ref172] Glennon R. A. Serotonin receptors and site-selective agents. J. Physiol. Pharmacol. 1991, 42, 49–60. 1932772

[ref173] Glennon R. A. Strategies for the development of selective serotonergic agents. In: The Serotonin Receptors; Roth B. L., Ed.; Humana Press: Totowa, NJ, 2006; pp 91–142.

[ref174] Glennon R. A. Site-selective serotonin agonists as discriminative stimuli. Psychopharmacol. Ser. 1988, 4, 15–31. 10.1007/978-3-642-73223-2_2. 3293039

[ref175] Glennon R. A.; Rosecrans J. A.; Young R. Behavioral properties of psychoactive phenylisopropylamines in rats. Eur. J. Pharmacol. 1981, 76, 353–360. 10.1016/0014-2999(81)90106-0. 7327208

[ref176] Spencer D. G. Jr.; Glaser T.; Traber J. Serotonin receptor subtype mediation of the interoceptive discriminative stimuli induced by 5-methoxy-*N,N*-dimethyltryptamine. Psychopharmacology (Berl.) 1987, 93, 158–166. 10.1007/BF00179927. 3122248

[ref177] Glennon R. A.; De Vry J.; Spencer D. G. Jr.; Glaser T. Stimulus properties of tiflucarbine: a novel antidepressant agent. Pharmacol., Biochem. Behav. 1990, 37, 769–771. 10.1016/0091-3057(90)90561-U. 2151199

[ref178] Glennon R. A. Discriminative stimulus properties of the 5-HT_1A_ agonist 8-hydroxy-2-(di-n-propylamino)tetralin (8-OH DPAT). Pharmacol., Biochem. Behav. 1986, 25, 135–139. 10.1016/0091-3057(86)90243-1. 2944129

[ref179] Glennon R. A. Site-selective serotonin agonists as discriminative stimuli. In: Transduction Mechanisms of Drug Stimuli; Colpaert F. C., Balster R. L., Eds.; Springer-Verlag: Berlin, 1988; pp 16–31. 10.1007/978-3-642-73223-2_2 3293039

[ref180] Tricklebank M. D.; Neill J.; Kidd E. J.; Fozard J. R. Mediation of the discriminative stimulus properties of 8-hydroxy-2-(di-n-propylamino) tetralin (8-OH-DPAT) by the putative 5-HT_1A_ receptor. Eur. J. Pharmacol. 1987, 133, 47–56. 10.1016/0014-2999(87)90204-4. 2881789

[ref181] Fozard J. R.; Kidd E. J.; Neill J.; Tricklebank M. Further characterization of the discriminative stimulus induced by 8-hydroxy-2-(di-n-propylamino)-tetralin in rats. Br. J. Pharmacol. 1986, 88 (Suppl), 371P.

[ref182] Cunningham K. A.; Appel J. B. Neuropharmacological reassessment of the discriminative stimulus properties of d-lysergic acid diethylamide (LSD). Psychopharmacology (Berl.) 1987, 91, 67–73. 10.1007/BF00690929. 3103161

[ref183] Reissig C. J.; Eckler J. R.; Rabin R. A.; Winter J. C. The 5-HT_1A_ receptor and the stimulus effects of LSD in the rat. Psychopharmacology (Berl.) 2005, 182, 197–204. 10.1007/s00213-005-0068-6. 16025319 PMC1266303

[ref184] Leysen J. E.; Awouters F.; Kennis L.; Laduron P. M.; Vandenberk J.; Janssen P. A. Receptor binding profile of R 41 468, a novel antagonist at 5-HT_2_ receptors. Life Sci. 1981, 28, 1015–1022. 10.1016/0024-3205(81)90747-5. 6261070

[ref185] Leysen J. E.; Niemegeers C. J.; Van Nueten J. M.; Laduron P. M. [^3^H]Ketanserin (R 41 468), a selective ^3^H-ligand for serotonin2 receptor binding sites. Binding properties, brain distribution, and functional role. Mol. Pharmacol. 1981, 21, 301–314. 7099138

[ref186] Colpaert F. C.; Niemegeers C. J. E.; Janssen P. A. J. A drug discrimination analysis of lysergic acid diethylamide (LSD): In vivo agonist and antagonist effects of purported 5-hydroxytryptamine antagonists and pirenperone, a LSD antagonist. J. Pharmacol. Exp. Ther. 1982, 22, 206–214. 7062283

[ref187] Glennon R. A.; Young R.; Rosecrans J. A. Antagonism of the effects of the hallucinogen DOM and the purported 5-HT agonist quipazine by 5-HT_2_ antagonists. Eur. J. Pharmacol. 1983, 91, 189–196. 10.1016/0014-2999(83)90464-8. 6617740

[ref188] Li J.-L.; Gerak L. R.; France C. P. Drug discrimination studies in Rhesus monkeys: drug dependence and withdrawal. In: Drug Discrimination: Applications to Medicinal Chemistry and Drug Studies; Glennon R. A., Young R., Eds.; John Wiley and Sons: Hoboken, NJ, 2011; pp 417–430.

[ref189] Li J.-X.; Rice K. C.; France C. P. Discriminative stimulus effects of 1-(2,5-dimethoxy-4-methylphenyl)-2-aminopropane in rhesus monkeys. J. Pharmacol. Exp. Ther. 2008, 324, 827–833. 10.1124/jpet.107.130625. 17993605

[ref190] Ismaiel A. M.; De Los Angeles J.; Teitler M.; Ingher S.; Glennon R. A. Antagonism of 1-(2,5-dimethoxy-4-methylphenyl)-2-aminopropane stimulus with a newly identified 5-HT_2_- versus 5-HT_1C_-selective antagonist. J. Med. Chem. 1993, 36, 2519–2525. 10.1021/jm00069a010. 8355253

[ref191] Fiorella D.; Rabin R. A.; Winter J. C. The role of the 5-HT_2A_ and 5-HT_2C_ receptors in the stimulus effects of hallucinogenic drugs I: Antagonist correlation analysis. Psychopharmacology 1995, 121, 347–356. 10.1007/BF02246074. 8584617

[ref192] Rasmussen K.; Glennon R. A.; Aghajanian G. K. Phenethylamine hallucinogens in the locus coeruleus: potency of action correlates with rank order of 5-HT_2_ binding affinity. Eur. J. Pharmacol. 1986, 132, 79–82. 10.1016/0014-2999(86)90014-2. 3816969

[ref193] Marek G. J.; Aghajanian G. K. Indoleamine and the phenethylamine hallucinogens: mechanisms of psychotomimetic action. Drug Alcohol Depend. 1998, 51, 189–198. 10.1016/S0376-8716(98)00076-3. 9716940

[ref194] Minnema D.; Krynock G.; Young R.; Glennon R. A.; Rosecrans J. LSD as a discriminative stimulus: role of dorsal raphe nucleus. Subst. Alcohol Actions Misuse 1980, 1, 29–34. 7323940

[ref195] Hirschhorn I. D.; Hayes R. L.; Rosecrans J. A. Discriminative control of behavior by electrical stimulation of the dorsal raphe nucleus: generalization to lysergic acid diethylamide (LSD). Brain Res. 1975, 86, 134–138. 10.1016/0006-8993(75)90645-9. 1115989

[ref196] Mokler D. J.; Dixon M.; Stambaugh L. Electrical stimulation of the dorsal raphe nucleus as a discriminative stimulus: generalization to (±)-DOI. Pharmacol., Biochem. Behav. 1994, 48, 1041–1045. 10.1016/0091-3057(94)90218-6. 7972283

[ref197] Glennon R. A.; Titeler M.; Seggel M. R.; Lyon R. A. N-Methyl derivatives of the 5-HT_2_ agonist 1-(4-bromo-2,5-dimethoxyphenyl)-2-aminopropane. J. Med. Chem. 1987, 30, 930–932. 10.1021/jm00388a032. 3572981

[ref198] Glennon R. A.; Young R. Drug discrimination and mechanisms of drug action. In: Drug Discrimination: Applications to Medicinal Chemistry and Drug Studies; Glennon R. A., Young R., Eds.; John Wiley and Sons: Hoboken, NJ, 2011; pp 184–216.

[ref199] Glennon R. A. Discriminative stimulus properties of the serotonergic agent 1-(2,5-dimethoxy-4-iodophenyl)-2-aminopropane (DOI). Life Sci. 1986, 39, 825–830. 10.1016/0024-3205(86)90461-3. 2943960

[ref200] Schreiber R.; Brocco M.; Millan M. J. Blockade of the discriminative stimulus effects of DOI by MDL 100,907 and the ’atypical’ antipsychotics, clozapine and risperidone. Eur. J. Pharmacol. 1994, 264, 99–102. 10.1016/0014-2999(94)90643-2. 7530204

[ref201] Chojnacka-Wójcik E.; Kłodzińska A. Involvement of 5-hydroxytryptamine 2A (5-HT_2A_) receptors in the mediation of the discriminative stimulus properties of (±)DOI in rats. Polym. J. Pharmacol. 1997, 49, 299–304. 9566028

[ref202] Smith R. L.; Barrett R. J.; Sanders-Bush E. Mechanism of tolerance development to 2,5-dimethoxy-4-iodoamphetamine in rats: down-regulation of the 5-HT_2A_, but not 5-HT_2C_, receptor. Psychopharmacology (Berl.) 1999, 144, 248–254. 10.1007/s002130051000. 10435391

[ref203] Luethi D.; Liechti M. E. Monoamine transporter and receptor interaction profiles in vitro predict reported human doses of novel psychoactive stimulants and psychedelics. Int. J. Neuropsychopharmacol. 2018, 21, 926–931. 10.1093/ijnp/pyy047. 29850881 PMC6165951

[ref204] Marona-Lewicka D.; Chemel B. R.; Nichols D. E. Dopamine D4 receptor involvement in the discriminative stimulus effects in rats of LSD, but not the phenethylamine hallucinogen DOI. Psychopharmacology (Berl.) 2009, 203, 265–277. 10.1007/s00213-008-1238-0. 18604600

[ref205] Glennon R. A.; Titeler M.; Lyon R. A. A preliminary investigation of the psychoactive agent 4-bromo-2,5-dimethoxyphenethylamine: a potential drug of abuse. Pharmacol., Biochem. Behav. 1988, 30, 597–601. 10.1016/0091-3057(88)90071-8. 3211969

[ref206] Glennon R. A.; Kier L. B.; Shulgin A. T. Molecular connectivity analysis of hallucinogenic mescaline analogs. J. Pharm. Sci. 1979, 68, 906–907. 10.1002/jps.2600680733. 458614

[ref207] Braun U.; Braun G.; Jacob P. III; Nichols D. E.; Shulgin A. T. Mescaline analogs: substituents at the 4-position. In: QuaSAR: Quantitative Structure Activity Relationships of Analgesics, Narcotic Antagonists, and Hallucinogens; Barnett G., Trsic M., Willette R. E., Eds.; National Institute on Drug Abuse: Washington D.C., 1978; pp 27–37.

[ref208] Braden M. R.; Parrish J. C.; Naylor J. C.; Nichols D. E. Molecular interaction of serotonin 5-HT_2A_ receptor residues Phe339(6.51) and Phe340(6.52) with superpotent N-benzyl phenethylamine agonists. Mol. Pharmacol. 2006, 70, 1956–1964. 10.1124/mol.106.028720. 17000863

[ref209] Johnson M. P.; Mathis C. A.; Shulgin A. T.; Hoffman A. J.; Nichols D. E. [^125^I]-2-(2,5-Dimethoxy-4-iodophenyl)aminoethane ([^125^I]-2C-I) as a label for the 5-HT_2_ receptor in rat frontal cortex. Pharmacol., Biochem. Behav. 1990, 35, 211–217. 10.1016/0091-3057(90)90228-A. 2315361

[ref210] Braun U.; Shulgin A. T.; Braun G.; Sargent T. 3rd Synthesis and body distribution of several iodine-131 labeled centrally acting drugs. J. Med. Chem. 1977, 20, 1543–1546. 10.1021/jm00222a001. 592317

[ref211] Moya P. R.; Berg K. A.; Gutiérrez-Hernandez M. A.; Sáez-Briones P.; Reyes-Parada M.; Cassels B. K.; Clarke W. P. Functional selectivity of hallucinogenic phenethylamine and phenylisopropylamine derivatives at human 5-hydroxytryptamine (5-HT)2A and 5-HT2C receptors. J. Pharmacol. Exp. Ther. 2007, 321, 1054–1061. 10.1124/jpet.106.117507. 17337633

[ref212] Acuña-Castillo C.; Villalobos C.; Moya P. R.; Sáez P.; Cassels B. K.; Huidobro-Toro J. P. Differences in potency and efficacy of a series of phenylisopropylamine/ phenylethylamine pairs at 5-HT(2A) and 5-HT(2C) receptors. Br. J. Pharmacol. 2002, 136, 510–519. 10.1038/sj.bjp.0704747. 12055129 PMC1573376

[ref213] Schmaljohann J.; Becherer A.; Kletter K.; Gündisch D. Radiosynthesis and *in vitro* evaluation of the 5HT_2_ receptor ligand [N-^11^C-methyl]-1-[2,5-dimethoxy-4-iodophenyl]-2-methylaminopropane for PET″. Radiochim. Acta 2004, 92, 345–348. 10.1524/ract.92.4.345.35592.

[ref214] Standridge R. T.; Howell H. G.; Tilson H. A.; Chamberlain J. H.; Holava H. M.; Gylys J. A.; Partyka R. A.; Shulgin A. T. Phenylalkylamines with potential psychotherapeutic utility. 2. Nuclear substituted 2-amino-1-phenylbutanes. J. Med. Chem. 1980, 23, 154–162. 10.1021/jm00176a010. 7359529

[ref215] Winter J. C. Effects of the phenethylamine derivatives, BL-3912, fenfluramine, and Sch-12679, in rats trained with LSD as a discriminative stimulus. Psychopharmacology 1980, 68, 159–162. 10.1007/BF00432134. 6776559

[ref216] Cunningham M. J.; Bock H. A.; Serrano I. C.; Bechand B.; Vidyadhara D. J.; Bonniwell E. M.; Lankri D.; Duggan P.; Nazarova A. L.; Cao A. B.; Calkins M. M.; Khirsariya P.; Hwu C.; Katritch V.; Chandra S. S.; McCorvy J. D.; Sames D. Pharmacological mechanism of the non-hallucinogenic 5-HT_2A_ agonist Ariadne and analogs. ACS Chem. Neurosci. 2023, 14, 119–135. 10.1021/acschemneuro.2c00597. 36521179 PMC10147382

[ref217] Ariëns E. J.; Simonis A. M. A molecular basis for drug action. J. Pharm. Pharmacol. 2011, 16, 137–157. 10.1111/j.2042-7158.1964.tb07437.x. 14163978

[ref218] Ariëns E. J. Agonists and antagonists – their structural relationship. In: Drug Design. I.; Ariëns E. J., Ed.; Academic Press: New York, 1971; pp 162–193.

[ref219] Glennon R. A.; Dukat M.; el-Bermawy M.; Law H.; De los Angeles J.; Teitler M.; King A.; Herrick-Davis K. Influence of amine substituents on 5-HT_2A_ versus 5-HT_2C_ binding of phenylalkyl- and indolylalkylamines. J. Med. Chem. 1994, 37, 1929–1935. 10.1021/jm00039a004. 8027974

[ref220] Nichols D. E.; Frescas S. P.; Chemel B. R.; Rehder K. S.; Zhong D.; Lewin A. H. High specific activity tritium-labeled *N*-(2-methoxybenzyl)-2,5-dimethoxy-4-iodophenethylamine (INBMeO): a high-affinity 5-HT_2A_ receptor-selective agonist radioligand. Bioorg. Med. Chem. 2008, 16, 6116–6123. 10.1016/j.bmc.2008.04.050. 18468904 PMC2719953

[ref221] Monte A. P.; Marona-Lewicka D.; Parker M. A.; Wainscott D. B.; Nelson D. L.; Nichols D. E. Dihydrobenzofuran analogues of hallucinogens. 3. Models of 4-substituted (2,5-dimethoxyphenyl)alkylamine derivatives with rigidified methoxy groups. J. Med. Chem. 1996, 39, 2953–2961. 10.1021/jm960199j. 8709129

[ref222] Parker M. A.; Marona-Lewicka D.; Lucaites V. L.; Nelson D. L.; Nichols D. E. A novel (benzodifuranyl)aminoalkane with extremely potent activity at the 5-HT_2A_ receptor. J. Med. Chem. 1998, 41, 5148–5149. 10.1021/jm9803525. 9857084

[ref223] Chambers J. J.; Kurrasch-Orbaugh D. M.; Parker M. A.; Nichols D. E. Enantiospecific synthesis and pharmacological evaluation of a series of super-potent, conformationally restricted 5-HT(2A/2C) receptor agonists. J. Med. Chem. 2001, 44, 1003–1010. 10.1021/jm000491y. 11300881

[ref224] Hansen M.; Phonekeo K.; Paine J. S.; Leth-Petersen S.; Begtrup M.; Bräuner-Osborne H.; Kristensen J. L. Synthesis and structure-activity relationships of N-benzyl phenethylamines as 5-HT_2A/2C_ agonists. ACS Chem. Neurosci. 2014, 5, 243–249. 10.1021/cn400216u. 24397362 PMC3963123

[ref225] Shulgin A. T. DOI as Hallucinogen: psychotomimetic drugs: structure-activity relationships. In: Handbook of Psychopharmacology, Vol. 2; Snyder S. H., Iversen L. L., Iversen S. D., Eds.; Plenum Press: New York, 1978; pp 243–333.

[ref226] Herian M.; Wojtas A.; Maćkowiak M.; Wawrzczak-Bargiela A.; Solarz A.; Bysiek A.; Madej K.; Gołembiowska K. Neurotoxicological profile of the hallucinogenic compound 25I-NBOMe. Sci. Rep. 2022, 12, 2939 10.1038/s41598-022-07069-8. 35190675 PMC8861095

[ref227] Pelletier R.; Gicquel T.; Carvelli J.; Balaz P.; Pelissier-Alicot A. L.; Morel I.; Bottinelli C.; Solas C.; Le Daré B.; Fabresse N. Severe 25E-NBOH intoxication associated with MDPHP intake: a case report, metabolism study, and literature review. Int. J. Legal Med. 2024, 138, 815 10.1007/s00414-023-03151-6. 38117418

[ref228] Schechter L. E.; Simansky K. J. 1-(2,5-Dimethoxy-4-iodophenyl)-2-aminopropane (DOI) exerts an anorexic action that is blocked by 5-HT_2_ antagonists in rats. Psychopharmacology (Berl.) 1988, 94, 342–346. 10.1007/BF00174687. 3128809

[ref229] Simansky K. J.; Vaidya A. H. Behavioral mechanisms for the anorectic action of the serotonin (5-HT) uptake inhibitor sertraline in rats: comparison with directly acting 5-HT agonists. Brain Res. Bull. 1990, 25, 953–960. 10.1016/0361-9230(90)90194-5. 2149668

[ref230] Aulakh C. S.; Hill J. L.; Yoney H. T.; Murphy D. L. Evidence for involvement of 5-HT_1C_ and 5-HT_2_ receptors in the food intake suppressant effects of 1-(2,5-dimethoxy-4-iodophenyl)-2-aminopropane (DOI). Psychopharmacology (Berl.) 1992, 109, 444–448. 10.1007/BF02247721. 1365860

[ref231] Bovetto S.; Richard D. Functional assessment of the 5-HT 1A-, 1B-, 2A/2C-, and 3-receptor subtypes on food intake and metabolic rate in rats. Am. J. Physiol. 1995, 268 (1 Pt 2), R14–R20. 10.1152/ajpregu.1995.268.1.R14. 7840314

[ref232] Aulakh C. S.; Mazzola-Pomietto P.; Hulihan-Giblin B. A.; Murphy D. L. Lack of cross-tolerance for hypophagia induced by DOI versus m-CPP suggests separate mediation by 5-HT_2A_ and 5-HT_2C_ receptors, respectively. Neuropsychopharmacology 1995, 13, 1–8. 10.1016/0893-133X(94)00127-L. 8526967

[ref233] Njung’e K.; Handley S. L. Effects of 5-HT uptake inhibitors, agonists and antagonists on the burying of harmless objects by mice; a putative test for anxiolytic agents. Br. J. Pharmacol. 1991, 104, 105–112. 10.1111/j.1476-5381.1991.tb12392.x. 1686200 PMC1908295

[ref234] Odland A. U.; Jessen L.; Kristensen J. L.; Fitzpatrick C. M.; Andreasen J. T. The 5-hydroxytryptamine 2A receptor agonists DOI and 25CN-NBOH decrease marble burying and reverse 8-OH-DPAT-induced deficit in spontaneous alternation. Neuropharmacology 2021, 183, 107838 10.1016/j.neuropharm.2019.107838. 31693871

[ref235] Odland A. U.; Kristensen J. L.; Andreasen J. T. Investigating the role of 5-HT_2A_ and 5-HT_2C_ receptor activation in the effects of psilocybin, DOI, and citalopram on marble burying in mice. Behav. Brain Res. 2021, 401, 113093 10.1016/j.bbr.2020.113093. 33359368

[ref236] Onaivi E. S.; Bishop-Robinson C.; Darmani N. A.; Sanders-Bush E. Behavioral effects of (±)-1-(2,5-dimethoxy-4-iodophenyl)-2-aminopropane, (DOI) in the elevated plus-maze test. Life Sci. 1995, 57, 2455–2466. 10.1016/0024-3205(95)02242-9. 8847967

[ref237] Serres F.; Azorin J. M.; Valli M.; Jeanningros R. Evidence for an increase in functional platelet 5-HT_2A_ receptors in depressed patients using the new ligand [^125^I]-DOI. Eur. Psychiat. 1999, 14, 451–457. 10.1016/S0924-9338(99)00222-9. 10683631

[ref238] Nic Dhonnchadha B. A.; Bourin M.; Hascoët M. Anxiolytic-like effects of 5-HT_2_ ligands on three mouse models of anxiety. Behav. Brain Res. 2003, 140, 203–214. 10.1016/S0166-4328(02)00311-X. 12644293

[ref239] Massé F.; Hascoët M.; Dailly E.; Bourin M. Effect of noradrenergic system on the anxiolytic-like effect of DOI (5-HT_2A/2C_ agonists) in the four-plate test. Psychopharmacology (Berl.) 2006, 183, 471–481. 10.1007/s00213-005-0220-3. 16307296

[ref240] Masse F.; Petit-Demouliere B.; Dubois I.; Hascoët M.; Bourin M. Anxiolytic-like effects of DOI microinjections into the hippocampus (but not the amygdala nor the PAG) in the mice four plates test. Behav. Brain Res. 2007, 188, 291–297. 10.1016/j.bbr.2007.11.005. 18155304

[ref241] Li Q.; Luo T.; Jiang X.; Wang J. Anxiolytic effects of 5-HT_1A_ receptors and anxiogenic effects of 5-HT_2C_ receptors in the amygdala of mice. Neuropharmacology 2012, 62, 474–684. 10.1016/j.neuropharm.2011.09.002. 21925519 PMC3196065

[ref242] Egashira N.; Okuno R.; Shirakawa A.; Nagao M.; Mishima K.; Iwasaki K.; Oishi R.; Fujiwara M. Role of 5-hydroxytryptamine2C receptors in marble-burying behavior in mice. Biol. Pharm. Bull. 2012 35, 376–379. 10.1248/bpb.35.376. PMID: 22382324. 22382324

[ref243] Muguruza C.; Moreno J. L.; Umali A.; Callado L. F.; Meana J. J.; González-Maeso J. Dysregulated 5-HT(2A) receptor binding in postmortem frontal cortex of schizophrenic subjects. Eur. Neuropsychopharmacol. 2013, 23, 852–864. 10.1016/j.euroneuro.2012.10.006. 23176747 PMC3586752

[ref244] Santini M. A.; Ratner C.; Aznar S.; Klein A. B.; Knudsen G. M.; Mikkelsen J. D. Enhanced prefrontal serotonin 2A receptor signaling in the subchronic phencyclidine mouse model of schizophrenia. J. Neurosci. Res. 2013, 91, 634–641. 10.1002/jnr.23198. 23404493

[ref245] Takao K.; Nagatani T.; Kitamura Y.; Kawasaki K.; Hayakawa H.; Yamawaki S. Chronic forced swim stress of rats increases frontal cortical 5-HT receptors and the wet-dog shakes they mediate, but not frontal cortical beta-adrenoceptors. Eur. J. Pharmacol. 1995, 294, 721–726. 10.1016/0014-2999(95)00620-6. 8750738

[ref246] Redrobe J. P.; Bourin M. Partial role of 5-HT_2_ and 5-HT_3_ receptors in the activity of antidepressants in the mouse forced swimming test. Eur. J. Pharmacol. 1997, 325, 129–135. 10.1016/S0014-2999(97)00115-5. 9163559

[ref247] Johnson R. G.; Stevens K. E.; Rose G. M. 5-Hydroxytryptamine2 receptors modulate auditory filtering in the rat. J. Pharmacol. Exp. Ther. 1998, 285, 643–650. 9580608

[ref248] McCall R. B.; Patel B. N.; Harris L. T. Effects of serotonin1 and serotonin2 receptor agonists and antagonists on blood pressure, heart rate and sympathetic nerve activity. J. Pharmacol. Exp. Ther. 1987, 242, 1152–1159. 2958619

[ref249] Dabiré H.; Chaouche-Teyara K.; Cherqui C.; Fournier B.; Laubie M.; Schmitt H. Characterization of DOI, a putative 5-HT_2_ receptor agonist in the rat. Eur. J. Pharmacol. 1989, 168, 369–374. 10.1016/0014-2999(89)90799-1. 2583242

[ref250] Alper R. H. Hemodynamic and renin responses to (±)-DOI, a selective 5-HT_2_ receptor agonist, in conscious rats. Eur. J. Pharmacol. 1990, 175, 323–332. 10.1016/0014-2999(90)90571-M. 2182326

[ref251] Dave K. D.; Quinn J. L.; Harvey J. A.; Aloyo V. J. Role of central 5-HT_2_ receptors in mediating head bobs and body shakes in the rabbit. Pharmacol., Biochem. Behav. 2004, 77, 623–629. 10.1016/j.pbb.2003.12.017. 15006475

[ref252] Schindler E. A.; Dave K. D.; Smolock E. M.; Aloyo V. J.; Harvey J. A. Serotonergic and dopaminergic distinctions in the behavioral pharmacology of (±)-1-(2,5-dimethoxy-4-iodophenyl)-2-aminopropane (DOI) and lysergic acid diethylamide (LSD). Pharmacol., Biochem. Behav. 2012, 101, 69–76. 10.1016/j.pbb.2011.12.002. 22197710 PMC3272148

[ref253] Schindler E. A.; Harvey J. A.; Aloyo V. J. Phospholipase C mediates (±)-1-(2,5-dimethoxy-4-iodophenyl)-2-aminopropane (DOI)-, but not lysergic acid diethylamide (LSD)-elicited head bobs in rabbit medial prefrontal cortex. Brain Res. 2013, 1491, 98–108. 10.1016/j.brainres.2012.10.057. 23123701 PMC3559188

[ref254] Vaidya V. A.; Marek G. J.; Aghajanian G. K.; Duman R. S. 5-HT_2A_ receptor-mediated regulation of brain-derived neurotrophic factor mRNA in the hippocampus and the neocortex. J. Neurosci. 1997, 17, 2785–2795. 10.1523/JNEUROSCI.17-08-02785.1997. 9092600 PMC6573109

[ref255] Gewirtz J. C.; Chen A. C.; Terwilliger R.; Duman R. C.; Marek G. J. Modulation of DOI-induced increases in cortical BDNF expression by group II mGlu receptors. Pharmacol., Biochem. Behav. 2002, 73, 317–326. 10.1016/S0091-3057(02)00844-4. 12117585

[ref256] Klein A. B.; Santini M. A.; Aznar S.; Knudsen G. M.; Rios M. Changes in 5-HT_2A_-mediated behavior and 5-HT_2A_- and 5-HT_1A_ receptor binding and expression in conditional brain-derived neurotrophic factor knock-out mice. Neuroscience 2010, 169, 1007–1016. 10.1016/j.neuroscience.2010.05.054. 20576498 PMC2914121

[ref257] Wischhof L.; Koch M. Pre-treatment with the mGlu2/3 receptor agonist LY379268 attenuates DOI-induced impulsive responding and regional c-Fos protein expression. Psychopharmacology (Berl.) 2012, 219, 387–400. 10.1007/s00213-011-2441-y. 21863235

[ref258] Tsybko A. S.; Ilchibaeva T. V.; Filimonova E. A.; Eremin D. V.; Popova N. K.; Naumenko V. S. The chronic treatment with 5-HT_2A_ receptor agonists affects the behavior and the BDNF system in mice. Neurochem. Res. 2020, 45, 3059–3075. 10.1007/s11064-020-03153-5. 33095437

[ref259] Nagayama H.; Lu J. Q. Circadian rhythm in the responsiveness of central 5-HT_2A_ receptor to DOI in rats. Psychopharmacology (Berl.) 1996, 127, 113–116. 10.1007/BF02805983. 8888376

[ref260] Filip M.; Bubar M. J.; Cunningham K. A. Contribution of serotonin (5-hydroxytryptamine; 5-HT) 5-HT_2_ receptor subtypes to the hyperlocomotor effects of cocaine: acute and chronic pharmacological analyses. J. Pharmacol. Exp. Ther. 2004, 310, 1246–1254. 10.1124/jpet.104.068841. 15131246

[ref261] Leah Salinsky L.; Merritt C. R.; Anastasio N. C.; Cunningham K. A. *R*-(−)2,5-Dimethoxy-4-iodoamphetamine [(−)-DOI] decreases cocaine demand in a 5-HT_2A_R-mediated manner. J. Pharmacol. Exp. Ther. 2023, 385 (S3), 3 10.1124/jpet.122.278820.

[ref262] Odland A. U.; Kristensen J. L.; Andreasen J. T. Investigating the role of 5-HT_2A_ and 5-HT_2C_ receptor activation in the effects of psilocybin, DOI, and citalopram on marble burying in mice. Behav. Brain Res. 2021, 401, 113093 10.1016/j.bbr.2020.113093. 33359368

[ref263] Berendsen H. H.; Broekkamp C. L. Comparison of stimulus properties of fluoxetine and 5-HT receptor agonists in a conditioned taste aversion procedure. Eur. J. Pharmacol. 1994, 253, 83–89. 10.1016/0014-2999(94)90760-9. 8013551

[ref264] Nash J. F. Jr.; Meltzer H. Y.; Gudelsky G. A. Selective cross-tolerance to 5-HT_1A_ and 5-HT_2_ receptor-mediated temperature and corticosterone responses. Pharmacol., Biochem. Behav. 1989, 33, 781–785. 10.1016/0091-3057(89)90470-X. 2533356

[ref265] Berendsen H. H.; Kester R. C.; Peeters B. W.; Broekkamp C. L. Modulation of 5-HT receptor subtype-mediated behaviours by corticosterone. Eur. J. Pharmacol. 1996, 308, 103–111. 10.1016/0014-2999(96)00286-5. 8840120

[ref266] Desouza L. A.; Benekareddy M.; Fanibunda S. E.; Mohammad F.; Janakiraman B.; Ghai U.; Gur T.; Blendy J. A.; Vaidya V. A. The hallucinogenic serotonin_2A_ receptor agonist, 2,5-dimethoxy-4-iodoamphetamine, promotes cAMP response element binding protein-dependent gene expression of specific plasticity-associated genes in the rodent neocortex. Front. Mol. Neurosci. 2021, 14, 790213 10.3389/fnmol.2021.790213. 35002622 PMC8739224

[ref267] Wright I. K.; Garratt J. C.; Marsden C. A. Effects of a selective 5-HT_2_ agonist, DOI, on 5-HT neuronal firing in the dorsal raphe nucleus and 5-HT release and metabolism in the frontal cortex. Br. J. Pharmacol. 1990, 99, 221–222. 10.1111/j.1476-5381.1990.tb14683.x. 1691671 PMC1917392

[ref268] Berquist M. D.; Fantegrossi W. E. Effects of 5-HT_2A_ receptor agonist 2,5-dimethoxy-4-iodoamphetamine on alcohol consumption in Long-Evans rats. Behav. Pharmacol. 2021, 32, 382–391. 10.1097/FBP.0000000000000628. 33595958 PMC8266736

[ref269] Arnt J.; Hyttel J. Facilitation of 8-OHDPAT-induced forepaw treading of rats by the 5-HT_2_ agonist DOI. Eur. J. Pharmacol. 1989, 161, 45–51. 10.1016/0014-2999(89)90178-7. 2524390

[ref270] Ashby C. R. Jr.; Edwards E.; Harkins K.; Wang R. Y. Effects of (±)-DOI on medial prefrontal cortical cells: a microiontophoretic study. Brain Res. 1989, 498, 393–396. 10.1016/0006-8993(89)91124-4. 2790491

[ref271] Molinaro G.; Traficante A.; Riozzi B.; Di Menna L.; Curto M.; Pallottino S.; Nicoletti F.; Bruno V.; Battaglia G. Activation of mGlu2/3 metabotropic glutamate receptors negatively regulates the stimulation of inositol phospholipid hydrolysis mediated by 5-hydroxytryptamine2A serotonin receptors in the frontal cortex of living mice. Mol. Pharmacol. 2009, 76, 379–387. 10.1124/mol.109.056580. 19439499

[ref272] Dursun S. M.; Handley S. L. Similarities in the pharmacology of spontaneous and DOI-induced head-shakes suggest 5HT_2A_ receptors are active under physiological conditions. Psychopharmacology (Berl.) 1996, 128, 198–205. 10.1007/s002130050125. 8956381

[ref273] Baudrie V.; Chaouloff F. Mechanisms involved in the hyperglycemic effect of the 5-HT_1C_/5-HT_2_ receptor agonist, DOI. Eur. J. Pharmacol. 1992, 213, 41–46. 10.1016/0014-2999(92)90230-2. 1499656

[ref274] Mazzola-Pomietto P.; Aulakh C. S.; Wozniak K. M.; Hill J. L.; Murphy D. L. Evidence that 1-(2,5-dimethoxy-4-iodophenyl)-2-aminopropane (DOI)-induced hyperthermia in rats is mediated by stimulation of 5-HT_2A_ receptors. Psychopharmacology (Berl.) 1995, 117, 193–199. 10.1007/BF02245187. 7753967

[ref275] Aulakh C. S.; Mazzola-Pomietto P.; Murphy D. L. Long-term antidepressant treatments alter 5-HT_2A_ and 5-HT_2C_ receptor-mediated hyperthermia in Fawn-Hooded rats. Eur. J. Pharmacol. 1995, 282, 65–70. 10.1016/0014-2999(95)00279-T. 7498290

[ref276] Yamada J.; Sugimoto Y.; Horisaka K. Serotonin2 (5-HT_2_) receptor agonist 1-(2,5-dimethoxy-4-iodophenyl)-2-aminopropane (DOI) inhibits chlorpromazine- and haloperidol-induced hypothermia in mice. Biol. Pharm. Bull. 1995, 18, 1580–1583. 10.1248/bpb.18.1580. 8593484

[ref277] Salmi P.; Ahlenius S. Evidence for functional interactions between 5-HT_1A_ and 5-HT_2A_ receptors in rat thermoregulatory mechanisms. Pharmacol. Toxicol. 1998, 82, 122–127. 10.1111/j.1600-0773.1998.tb01410.x. 9553989

[ref278] Flanagan T. W.; Nichols C. D. Psychedelics as anti-inflammatory agents. Int. Rev. Psychiatry. 2018, 30, 363–375. 10.1080/09540261.2018.1481827. 30102081

[ref279] Flanagan T. W.; Sebastian M. N.; Battaglia D. M.; Foster T. P.; Cormier S. A.; Nichols C. D. 5-HT_2_ receptor activation alleviates airway inflammation and structural remodeling in a chronic mouse asthma model. Life Sci. 2019, 236, 116790 10.1016/j.lfs.2019.116790. 31626791

[ref280] Jaster A. M.; Elder H.; Marsh S. A.; de la Fuente Revenga M.; Negus S. S.; González-Maeso J. Effects of the 5-HT_2A_ receptor antagonist volinanserin on head-twitch response and intracranial self-stimulation depression induced by different structural classes of psychedelics in rodents. Psychopharmacology 2022, 239, 1665–1677. 10.1007/s00213-022-06092-x. 35233648 PMC10055857

[ref281] May J. A.; McLaughlin M. A.; Sharif N. A.; Hellberg M. R.; Dean T. R. Evaluation of the ocular hypotensive response of serotonin 5-HT_1A_ and 5-HT_2_ receptor ligands in conscious ocular hypertensive cynomolgus monkeys. J. Pharmacol. Exp. Ther. 2003, 306, 301–309. 10.1124/jpet.103.049528. 12676887

[ref282] Krebs K. M.; Geyer M. A. Cross-tolerance studies of serotonin receptors involved in behavioral effects of LSD in rats. Psychopharmacology (Berl.) 1994, 113, 429–437. 10.1007/BF02245219. 7862855

[ref283] Plaznik A.; Stefanski R.; Palejko W.; Bidzinski A.; Kostowski W.; Jessa M.; Nazar M. Antidepressant treatment and limbic serotonergic mechanisms regulating rat locomotor activity. Pharmacol., Biochem. Behav. 1994, 48, 315–325. 10.1016/0091-3057(94)90533-9. 8090797

[ref284] Halberstadt A. L.; van der Heijden I.; Ruderman M. A.; Risbrough V. B.; Gingrich J. A.; Geyer M. A.; Powell S. B. 5-HT(2A) and 5-HT(2C) receptors exert opposing effects on locomotor activity in mice. Neuropsychopharmacology 2009, 34, 1958–1967. 10.1038/npp.2009.29. 19322172 PMC2697271

[ref285] Wing L. L.; Tapson G. S.; Geyer M. A. 5HT-2 mediation of acute behavioral effects of hallucinogens in rats. Psychopharmacology (Berl.) 1990, 100, 417–425. 10.1007/BF02244617. 2138338

[ref286] Krebs-Thomson K.; Paulus M. P.; Geyer M. A. Effects of hallucinogens on locomotor and investigatory activity and patterns: influence of 5-HT_2A_ and 5-HT_2C_ receptors. Neuropsychopharmacology 1998, 18, 339–351. 10.1016/S0893-133X(97)00164-4. 9536447

[ref287] Löscher W.; Witte U.; Fredow G.; Ganter M.; Bickhardt K. Pharmacodynamic effects of serotonin (5-HT) receptor ligands in pigs: stimulation of 5-HT_2_ receptors induces malignant hyperthermia. Naunyn-Schmiedebergs Arch. Pharmacol. 1990, 341, 483–493. 10.1007/BF00171727. 2118235

[ref288] Wappler F.; Roewer N.; Kochling A.; Scholz J.; Loscher W.; Steinfath M.; Schulte am Esch J. Effects of the serotonin_2_ receptor agonist DOI on skeletal muscle specimens from malignant hyperthermia-susceptible patients. Anesthesiology 1996, 84, 1280–1287. 10.1097/00000542-199606000-00002. 8669667

[ref289] Wappler F.; Scholz J.; Oppermann S.; von Richthofen V.; Steinfath M.; Schulte am Esch J. Ritanserin attenuates the in vitro effects of the 5-HT_2_ receptor agonist DOI on skeletal muscles from malignant hyperthermia-susceptible patients. J. Clin. Anesth. 1997, 9, 306–311. 10.1016/S0952-8180(97)00008-1. 9195354

[ref290] Pierce P. A.; Peroutka S. J. Antagonism of 5-hydroxytryptamine_2_ receptor-mediated phosphatidylinositol turnover by d-lysergic acid diethylamide. J. Pharmacol. Exp. Ther. 1988, 247, 918–925. 3204523

[ref291] Edwards E.; Ashby C. R. Jr.; Wang R. Y. (±)-1-(2,5-Dimethoxy-4-iodophenyl)-2-aminopropane (DOI) and alpha-methyl-5-HT: 5-HT_2_ receptor agonistic action on phosphatidylinositol metabolism in the rat fronto-cingulate and entorhinal cortex. Neuropharmacology 1992, 31, 615–621. 10.1016/0028-3908(92)90139-G. 1407401

[ref292] Seggel M. R.; Qureshi G. D.; Glennon R. A. Effect of 5-HT_2_-selective agonists on cat platelet aggregation. Life Sci. 1987, 41, 1077–1081. 10.1016/0024-3205(87)90624-2. 3613863

[ref293] Britt S. G.; Gonias S. L.; Sanders J. M.; Vandenberg S. R. Agonist and antagonist activities of arylpiperazines at human platelet serotonin_2_ receptors. J. Pharmacol. Exp. Ther. 1988, 247, 965–970. 3204525

[ref294] Himeno A.; Saavedra J. M. Human platelet [^125^I]R-DOI binding sites. Characterization by in vitro autoradiography. Neuropsychopharmacology 1990, 3, 25–32. 2306333

[ref295] Kagaya A.; Mikuni M.; Kusumi I.; Yamamoto H.; Takahashi K. Serotonin-induced acute desensitization of serotonin_2_ receptors in human platelets via a mechanism involving protein kinase C. J. Pharmacol. Exp. Ther. 1990, 255, 305–311. 2213562

[ref296] Pan J. T.; Tai M. H. Effects of ketanserin on DOI-, MCPP- and TRH-induced prolactin secretion in estrogen-treated rats. Life Sci. 1992, 51, 839–845. 10.1016/0024-3205(92)90611-R. 1522746

[ref297] Albinsson A.; Palazidou E.; Stephenson J.; Andersson G. Involvement of the 5-HT_2_ receptor in the 5-HT receptor-mediated stimulation of prolactin release. Eur. J. Pharmacol. 1994, 251, 157–161. 10.1016/0014-2999(94)90396-4. 8149973

[ref298] Fantegrossi W. E.; Woods J. H.; Winger G. Transient reinforcing effects of phenylisopropylamine and indolealkylamine hallucinogens in rhesus monkeys. Behav. Pharmacol. 2004, 15, 149–157. 10.1097/00008877-200403000-00007. 15096915

[ref299] Pranzatelli M. R. Evidence for involvement of 5-HT_2_ and 5-HT_1C_ receptors in the behavioral effects of the 5-HT agonist 1-(2,5-dimethoxy-4-iodophenylaminopropane)-2 (DOI). Neurosci. Lett. 1990, 115, 74–80. 10.1016/0304-3940(90)90520-J. 2216059

[ref300] Foreman M. M.; Hall J. L.; Love R. L. The role of the 5-HT_2_ receptor in the regulation of sexual performance of male rats. Life Sci. 1989, 45, 1263–1270. 10.1016/0024-3205(89)90128-8. 2811596

[ref301] Watson N. V.; Gorzalka B. B. Relation of spontaneous wet dog shakes and copulatory behavior in male rats. Pharmacol., Biochem. Behav. 1990, 37, 825–829. 10.1016/0091-3057(90)90569-4. 2093184

[ref302] Watson N. V.; Gorzalka B. B. DOI-induced inhibition of copulatory behavior in male rats: reversal by 5-HT_2_ antagonists. Pharmacol., Biochem. Behav. 1991, 39, 605–612. 10.1016/0091-3057(91)90135-O. 1784589

[ref303] Gonzalez M. I.; Farabollini F.; Albonetti E.; Wilson C. A. Interactions between 5-hydroxytryptamine (5-HT) and testosterone in the control of sexual and nonsexual behaviour in male and female rats. Pharmacol., Biochem. Behav. 1994, 47, 591–601. 10.1016/0091-3057(94)90164-3. 8208779

[ref304] Padoin M. J.; Lucion A. B. The effect of testosterone and DOI (1-(2,5-dimethoxy-4-iodophenyl)-2-aminopropane) on male sexual behavior of rats. Eur. J. Pharmacol. 1995, 277, 1–6. 10.1016/0014-2999(95)00021-C. 7635164

[ref305] Rössler A. S.; Bernabé J.; Denys P.; Alexandre L.; Giuliano F. Effect of the 5-HT receptor agonist DOI on female rat sexual behavior. J. Sex Med. 2006, 3, 432–441. 10.1111/j.1743-6109.2006.00240.x. 16681468

[ref306] Banerjee A. A.; Vaidya V. A. Differential signaling signatures evoked by DOI versus lisuride stimulation of the 5-HT_2A_ receptor. Biochem. Biophys. Res. Commun. 2020, 531, 609–614. 10.1016/j.bbrc.2020.08.022. 32814630

[ref307] Fone K. C.; Robinson A. J.; Marsden C. A. Characterization of the 5-HT receptor subtypes involved in the motor behaviours produced by intrathecal administration of 5-HT agonists in rats. Br. J. Pharmacol. 1991, 103, 1547–1555. 10.1111/j.1476-5381.1991.tb09825.x. 1832068 PMC1908369

[ref308] Eide P. K.; Hole K. Different role of 5-HT_1A_ and 5-HT_2_ receptors in spinal cord in the control of nociceptive responsiveness. Neuropharmacology 1991, 30, 727–731. 10.1016/0028-3908(91)90180-J. 1717872

[ref309] Kjørsvik A.; Tjølsen A.; Hole K. Activation of spinal serotonin(2A/2C) receptors augments nociceptive responses in the rat. Brain Res. 2001, 910, 179–181. 10.1016/S0006-8993(01)02652-X. 11489268

[ref310] Padich R. A.; McCloskey T. C.; Kehne J. H. () 5-HT modulation of auditory and visual sensorimotor gating: II. Effects of the 5-HT_2A_ antagonist MDL 100,907 on disruption of sound and light pre-pulse inhibition produced by 5-HT agonists in Wistar rats. Psychopharmacology (Berl.) 1996, 124, 107–116. 10.1007/BF02245610. 8935805

[ref311] Buckholtz N. S.; Zhou D. F.; Freedman D. X. Serotonin2 agonist administration down-regulates rat brain serotonin2 receptors. Life Sci. 1988, 42, 2439–2445. 10.1016/0024-3205(88)90342-6. 3374263

[ref312] McKenna D. J.; Nazarali A. J.; Himeno A.; Saavedra J. M. Chronic treatment with (±)DOI, a psychotomimetic 5-HT_2_ agonist, downregulates 5-HT_2_ receptors in rat brain. Neuropsychopharmacology 1989, 2, 81–87. 10.1016/0893-133X(89)90010-9. 2803482

[ref313] Takaba R.; Ibi D.; Yoshida K.; Hosomi E.; Kawase R.; Kitagawa H.; Goto H.; Achiwa M.; Mizutani K.; Maeda K.; González-Maeso J.; Kitagaki S.; Hiramatsu M. Ethopharmacological evaluation of antidepressant-like effect of serotonergic psychedelics in C57BL/6J male mice. Naunyn-Schmiedebergs Arch. Pharmacol. **2023** Oct 24. 10.1007/s00210-023-02778-x. PMID: 37874338. PMC12910574 37874338

[ref314] Lewis V.; Bonniwell E. M.; Lanham J. K.; Ghaffari A.; Sheshbaradaran H.; Cao A. B.; Calkins M. M.; Bautista-Carro M. A.; Arsenault E.; Telfer A.; Taghavi-Abkuh F. F.; Malcolm N. J.; El Sayegh F.; Abizaid A.; Schmid Y.; Morton K.; Halberstadt A. L.; Aguilar-Valles A.; McCorvy J. D. A non-hallucinogenic LSD analog with therapeutic potential for mood disorders. Cell Rep. 2023, 42, 112203 10.1016/j.celrep.2023.112203. 36884348 PMC10112881

[ref315] Cameron L. P.; Benetatos J.; Lewis V.; Bonniwell E. M.; Jaster A. M.; Moliner R.; Castrén E.; McCorvy J. D.; Palner M.; Aguilar-Valles A. Beyond the 5-HT_2A_ receptor: classic and nonclassic targets in psychedelic drug action. J. Neurosci. 2023, 43, 7472–7482. 10.1523/JNEUROSCI.1384-23.2023. 37940583 PMC10634557

[ref316] Wallach J.; Cao A.; Calkins M.; Heim A. J.; Lanham J. K.; Bonniwell E. M.; Hennessey J. J.; Bock H. A.; Anderson E. I.; Sherwood A. M.; Morris H.; de Klein R.; Klein A. K.; Cuccurazzu B.; Gamrat J.; Fannana T.; Zauhar R.; Halberstadt A. L.; McCorvy J. D. Identification of 5-HT_2A_ receptor signaling pathways responsible for psychedelic potential. Nat. Commun. 2023, 14, 8221 10.1038/s41467-023-44016-1. 38102107 PMC10724237

[ref317] Kaplan A. L.; Confair D. N.; Kim K.; Barros-Álvarez X.; Rodriguiz R. M.; Yang Y.; Kweon O. S.; Che T.; McCorvy J. D.; Kamber D. N.; Phelan J. P.; Martins L. C.; Pogorelov V. M.; DiBerto J. F.; Slocum S. T.; Huang X. P.; Kumar J. M.; Robertson M. J.; Panova O.; Seven A. B.; Wetsel A. Q.; Wetsel W. C.; Irwin J. J.; Skiniotis G.; Shoichet B. K.; Roth B. L.; Ellman J. A. Bespoke library docking for 5-HT_2A_ receptor agonists with antidepressant activity. Nature 2022, 610, 582–591. 10.1038/s41586-022-05258-z. 36171289 PMC9996387

[ref318] Cao D.; Yu J.; Wang H.; Luo Z.; Liu X.; He L.; Qi J.; Fan L.; Tang L.; Chen Z.; Li J.; Cheng J.; Wang S. Structure-based discovery of nonhallucinogenic psychedelic analogs. Science 2022, 375 (6579), 403–411. 10.1126/science.abl8615. 35084960

[ref319] de la Fuente Revenga M.; Zhu B.; Guevara C. A.; Naler L. B.; Saunders J. M.; Zhou Z.; Toneatti R.; Sierra S.; Wolstenholme J. T.; Beardsley P. M.; Huntley G. W.; Lu C.; González-Maeso J. Prolonged epigenomic and synaptic plasticity alterations following single exposure to a psychedelic in mice. Cell Rep. 2021, 37, 109836 10.1016/j.celrep.2021.109836. 34686347 PMC8582597

[ref320] Moliner R.; Girych M.; Brunello C. A.; Kovaleva V.; Biojone C.; Enkavi G.; Antenucci L.; Kot E. F.; Goncharuk S. A.; Kaurinkoski K.; Kuutti M.; Fred S. M.; Elsilä L. V.; Sakson S.; Cannarozzo C.; Diniz C. R. A. F.; Seiffert N.; Rubiolo A.; Haapaniemi H.; Meshi E.; Nagaeva E.; Öhman T.; Róg T.; Kankuri E.; Vilar M.; Varjosalo M.; Korpi E. R.; Permi P.; Mineev K. S.; Saarma M.; Vattulainen I.; Casarotto P. C.; Castrén E. Psychedelics promote plasticity by directly binding to BDNF receptor TrkB. Nat. Neurosci. 2023, 26, 1032–1041. 10.1038/s41593-023-01316-5. 37280397 PMC10244169

[ref321] Schedules of Controlled Substances: Placement of 2,5-dimethoxy-4-iodoamphetamine (DOI) and 2,5-dimethoxy-4-chloroamphetamine (DOC) in Schedule I. Federal Register 88 (Wednesday, December 13, 2023), 86278–86284. [FR Doc. 2023-27289].

